# A Path‐Based Approach for Detecting and Assessing Inconsistency in Network Meta‐Analysis

**DOI:** 10.1002/sim.70752

**Published:** 2026-10-15

**Authors:** Noosheen R. Tahmasebi, Annabel L. Davies, Theodoros Papakonstantinou, Gerta Rücker, Adriani Nikolakopoulou

**Affiliations:** ^1^ Institute for Medical Biometry and Statistics (IMBI) Faculty of Medicine and Medical Center, University of Freiburg Freiburg Germany; ^2^ Institute for Evidence in Medicine (IfEM) Faculty of Medicine and Medical Center, University of Freiburg Freiburg Germany; ^3^ Bristol Medical School University of Bristol Bristol UK; ^4^ Laboratory of Hygiene, Social and Preventive Medicine and Medical Statistics School of Medicine, Aristotle University of Thessaloniki Thessaloniki Greece

**Keywords:** incoherence, inconsistency, mixed‐treatment comparison, network meta‐analysis, paths

## Abstract

Network Meta‐Analysis (NMA) is a statistical technique for synthesizing evidence from various sources to compare multiple interventions. NMA relies on integrating both direct evidence from head‐to‐head comparisons and indirect evidence from different paths that link treatments through common comparators. A key aspect is evaluating consistency between direct and indirect sources. Existing methods to detect inconsistency, although widely used, have limitations. For example, they do not account for differences between indirect sources or cannot estimate inconsistency when direct evidence is absent. In this paper, we introduce a path‐based approach that explores all sources of evidence without separating direct and indirect. We introduce a measure based on the square of differences to quantitatively capture inconsistency, and propose a Netpath plot to visualize inconsistencies between various paths. Moreover, it enables the evaluation of inconsistency even for treatment comparisons lacking direct evidence as long as at least two independent paths of evidence are available. We provide an implementation of our path‐based method within the netmeta R package. Via application to fictional and real‐world examples, we show that our method is able to detect and visualize inconsistency between multiple paths of evidence that would otherwise be masked by considering all indirect sources together. The path‐based approach therefore provides a more comprehensive evaluation of inconsistency within a network of treatments.

**Abbreviations**
DBT: design‐by‐treatment
DoF: degree of freedom
(in)dir: (in)direct
NMA: network meta‐analysis
RCTs: randomized controlled trials

## 1 Introduction

In medical research, randomized controlled trials (RCTs) have been acknowledged as the most reliable method for comparing treatment options [1]. As the collection of available treatments expands, the potential pairwise comparisons grow drastically [2]. The assessment of treatment efficacy commonly relies on direct (dir) comparisons in randomized trials. Indirect (indir) comparisons arise when two treatments have undergone direct comparisons with at least one other common treatment [3]. A treatment comparison refers to the difference in outcome effects between two treatments, which can be derived from either direct head‐to‐head comparison (known as a contrast) or indirectly through common comparators in a network of studies. Network meta‐analysis (NMA) offers a more informed estimate by incorporating both direct and indirect evidence. Unlike traditional meta‐analysis, which uses only head‐to‐head trials, NMA combines data from multiple sources, allowing for comparisons between treatments not directly compared in trials, leading to more comprehensive and precise estimates [4].

The inconsistency of a certain treatment comparison is defined as the conflict between different sources of evidence on that comparison [3]. Typically, this pertains to the conflict between direct and indirect evidence and has been addressed using various terms, including incoherence [2]. The presence of inconsistency in the network of evidence undermines the reliability of conclusions drawn from NMA. Therefore, investigation of consistency is important for making robust, evidence‐based medical decisions and policy recommendations.

Inconsistency is currently assessed using methods that evaluate the agreement between direct and indirect evidence by statistically testing for discrepancies. To assess or detect inconsistency in a network, local methods focus on individual parts or subsets of the network, such as specific treatment contrasts or loops within the network. Global methods evaluate the overall consistency of the entire network, providing a broad view to detect inconsistencies that affect the data or model as a whole. However, both these approaches have limitations. Local methods are often computationally expensive, and can fail to detect more nuanced inconsistencies between different sources of indirect evidence. Moreover, these methods cannot assess the inconsistency on a comparison when there is no direct evidence available. To address these limitations, we propose a method based on the concept of ‘paths of evidence’ [5]. Rather than segregating evidence into direct and indirect sources, we treat each indirect estimate separately. In this framework, a direct comparison becomes just one of many possible paths between two treatments. We then employ the concept of inconsistency to judge the coherence among all the paths between two treatments of interest, providing a thorough assessment of inconsistency beyond just direct versus indirect comparisons.

In this article, we make two primary contributions: (i) we perform a concise review of established methods used to detect and assess inconsistency in NMA, and (ii) we propose a novel path‐based method to quantify the degree to which different sources of evidence agree when obtaining a specific network estimate. To illustrate and compare the different methods, we use a toy example of a network with four treatments and five observed comparisons.

In Section 2, we first explain the assumption of inconsistency, then we explore existing methods for detecting inconsistency including loop‐specific, [4] side‐splitting, [6] net heat plot, [7] and the global design‐by‐treatment (DBT) interaction model [8]. We explain when and how these methods fall short, and compare their performance via application to our toy example. In Section 3, we introduce the path framework and propose an inconsistency measure based on the square of differences. This measure aims to increase the accuracy and interpretability of inconsistency assessment. In Section 4, we apply our method to a real‐world example, demonstrating its implementation and the ability of the algorithm to effectively handle larger networks.

## 2 Consistency Assumption in NMA

Consider a network of N nodes with treatments {1,…,N}. There are M=N(N−1)/2 possible comparisons and E direct comparisons (edges). Let us take a network with three treatments, 1,2,3, and three comparisons, as illustrated in Figure 1. Each comparison ij is informed by $S_{ij}$ studies. These trials may also include more than two treatments (arms) i and j, which introduces additional statistical challenges. In multi‐arm studies, individual comparisons are positively correlated because they share a common control arm; this dependency does not arise in separate two‐arm trials [9]. Rücker et al. propose addressing this issue by splitting multi‐arm trials into separate two‐arm comparisons with appropriately adjusted variances [10]. Under this approach, for example, a three‐arm trial can be represented as three two‐arm comparisons, each with updated variance estimates.

**FIGURE 1:**
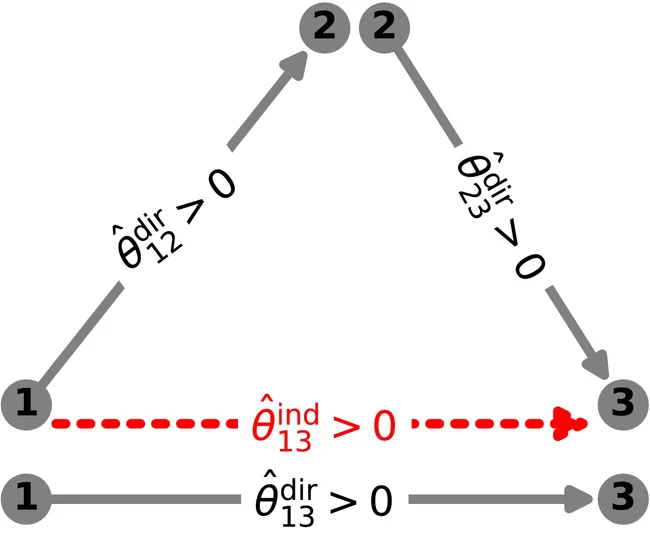
The simplest network with three treatments: 1, 2, and 3. The summary effect for each comparison, ${\hat{\theta }}_{ij}^{\text{dir}}$, is derived from a pairwise meta‐analysis based on data from $S_{ij}$ studies comparing the two treatments. The indirect estimate ${\hat{\theta }}_{13}^{\text{indir}}$ follows the logic that if the effect between 1‐2 is greater than 0, and the effect between 2‐3 is greater than 0, then the effect between 1‐3 should also be greater than 0.

Each ${\hat{\theta }}_{ij}^{\text{dir}}$ in Figure 1 represents the summary effect from a meta‐analysis of all trials comparing treatments i and j [11]. This structure is referred to as an aggregate model, where each comparison is characterized by a summary effect and its variance. The NMA estimate for a particular treatment comparison can then be expressed as a linear combination of all available estimates for that comparison. By integrating both direct and indirect evidence across all treatment pairs, NMA increases the precision of the estimated contrasts. For instance, in Figure 1, the NMA estimate for comparison 1‐3 incorporates both the direct evidence, ${\hat{\theta }}_{13}^{\text{dir}}$, and the indirect evidence, ${\hat{\theta }}_{13}^{\text{indir}}$. The indirect evidence in this case is given by ${\hat{\theta }}_{12}^{\text{dir}}+{\hat{\theta }}_{23}^{\text{dir}}$, as treatment 2 serves as a common comparator through which the effect between treatments 1 and 3 can be inferred indirectly.

The reliability of NMA is highly dependent on the consistency assumption, which requires different sources of evidence for a specific comparison to be in agreement. Here, we assume a beneficial outcome, such that a positive summary effect (${\hat{\theta }}_{ij}^{\text{dir}}>0$) indicates that treatment i is more effective than treatment j. If we observe ${\hat{\theta }}_{12}^{\text{dir}}>0$ (1 is better than 2) and ${\hat{\theta }}_{23}^{\text{dir}}>0$ (2 is better than 3), then the indirect estimate,

(1) $${\hat{\theta }}_{13}^{\text{indir}}={\hat{\theta }}_{12}^{\text{dir}}+{\hat{\theta }}_{23}^{\text{dir}},$$

implies that treatment 1 should be more effective than treatment 3 (${\hat{\theta }}_{13}^{\text{dir}}>0$). If the direct summary effect for 1‐3 is negative, ${\hat{\theta }}_{13}^{\text{dir}}<0$, the consistency assumption is violated. Such an inconsistency between the two sources of evidence jeopardizes the validity of NMA estimates in this network, as both sources (direct and indirect) are used to calculate the NMA estimate of 1‐3. In the following section, we first introduce a toy example which we use to briefly review several well‐established and commonly used methods, along with their limitations which motivated the development of the path‐based approach.

### 2.1 Motivating Example

Figure 2(a) shows an illustrative example network featuring four treatments (N=4, M=6) denoted by {1,2,3,4}, with E=5 direct comparisons: 1‐2, 1‐3, 1‐4, 2‐3, and 3‐4. For this toy example, we specify summary effect estimates ${\hat{\theta }}_{13}^{\text{dir}}=2$, ${\hat{\theta }}_{12}^{\text{dir}}={\hat{\theta }}_{23}^{\text{dir}}=0.5$, and ${\hat{\theta }}_{14}^{\text{dir}}={\hat{\theta }}_{43}^{\text{dir}}=1.5$. For our purposes, the choice of effect measure, whether it be risk ratio, mean difference, or any other metric, is not important. We assume all the studies have only two treatments, and the variances of all summary effects are equal. For the latter, we choose an arbitrary value of $\sigma_{ij}^{2}=0.3^{2}$.

**FIGURE 2:**
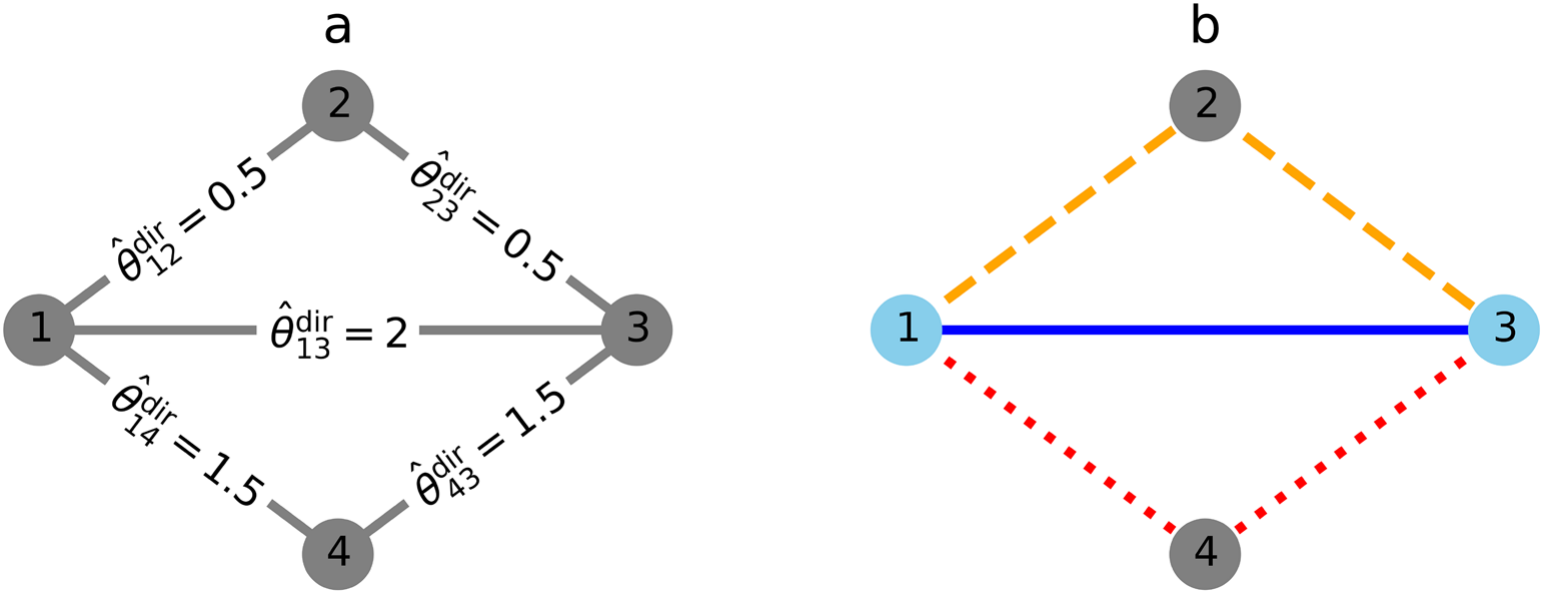
(a) A fictional example with four treatments {1,2,3,4}, and five direct comparisons 1‐2, 1‐3, 1‐4, 2‐3, and 3‐4. The ${\hat{\theta }}_{ij}^{\text{dir}}$ refers to the pairwise summary effect of $S_{ij}$ trials studying the comparison i vs j. (b) Three different sources of evidence for the comparison 1‐3. The treatments of interest are shown as blue nodes. The direct evidence is represented by a blue solid line, while the indirect evidences are represented by dashed orange and dotted red lines.

To elaborate on the concept of inconsistency, let us take the comparison 1‐3. For this comparison, we have a direct estimate ${\hat{\theta }}_{13}^{\text{dir}}=2$ and two indirect estimates, one via treatment 2 (1→2→3), and one via treatment 4 (1→4→3). In Figure 2(b), these are shown in dashed orange and dotted red respectively. The corresponding indirect estimates are ${\hat{\theta }}_{13}^{{\text{indir}}_{1}}={\hat{\theta }}_{12}^{\text{dir}}+{\hat{\theta }}_{23}^{\text{dir}}=1$ and ${\hat{\theta }}_{13}^{{\text{indir}}_{2}}={\hat{\theta }}_{14}^{\text{dir}}+{\hat{\theta }}_{43}^{\text{dir}}=3$. In this example, the three estimates of comparison 1‐3 disagree, ${\hat{\theta }}_{13}^{{\text{indir}}_{1}}\neq {\hat{\theta }}_{13}^{\text{dir}}\neq {\hat{\theta }}_{13}^{{\text{indir}}_{2}}$. This indicates some level of inconsistency. However, pooling the indirect estimates (with equal weights) leads to an overall indirect estimate

(2) $${\hat{\theta }}_{13}^{\text{indir(total)}}=\frac{1}{2}({\hat{\theta }}_{13}^{{\text{indir}}_{1}}+{\hat{\theta }}_{13}^{{\text{indir}}_{2}})=2,$$

which is equal to the direct estimate. We use this example to illustrate how different methods perform when inconsistencies between different indirect estimates are masked by the pooled indirect evidence.

### 2.2 Inconsistency Assessment, Conventional Methods

#### 2.2.1 Loop‐Specific

The loop‐specific method tests for inconsistency by examining closed loops of evidence in a network of treatments [12]. In this method, we test for differences between direct and indirect estimates in each loop. For a given loop ℓ, we perform a Z‐test on the difference,

(3) $$\omega_{\ell }=|{\hat{\theta }}_{ij}^{\text{dir}}-{\hat{\theta }}_{ij}^{\text{indir}}|,$$

where the null hypothesis assumes no inconsistency within the loop. The results from this test provide a p‐value that indicates the likelihood of inconsistency in a loop.

There are three loops in our toy example shown in Figure 2: ℓ=1: 1‐2‐3, ℓ=2: 1‐4‐3, and ℓ=3: 1‐2‐3‐4. Since the inconsistency measure $\omega_{\ell }$ is a property of the loop, it does not depend on which comparison is chosen as the direct estimate [12, 13]. For instance, in the first loop (ℓ=1), it makes no difference whether 1‐2, 1‐3, or 2‐3 is selected as the direct comparison: the total difference between the direct and indirect estimates will yield the same result. Following the loop‐specific approach, one gets the following results for these three loops

$$\begin{array}{ll} \omega_{1} & =|{\hat{\theta }}_{13}^{\text{(dir)}}-{\hat{\theta }}_{13}^{{\text{indir}}_{1}}|=|2-1|=1,\\ \omega_{2} & =|{\hat{\theta }}_{13}^{\text{(dir)}}-{\hat{\theta }}_{13}^{{\text{indir}}_{2}}|=|2-3|=1,\\ \omega_{3} & =|{\hat{\theta }}_{23}^{\text{(dir)}}-{\hat{\theta }}_{23}^{\text{indir}}|=|0.5-2.5|=2. \end{array}$$

We get the same results for ℓ=1 and ℓ=2 because the discrepancies between the direct estimate and each of the indirect estimates are the same. The p‐value for both loops is 0.05, and the third loop has a p‐value of 0.0008.

There are several limitations to the loop‐specific approach. First, this method performs poorly in the presence of multi‐arm trials [8]. Because multi‐arm trials are internally consistent, they are often excluded from the calculation of the inconsistency measure $\omega_{\ell }$. However, excluding available data is generally undesirable. In addition, the approach becomes increasingly complex as the number of loops grows, making it difficult to identify independent loops within larger networks and potentially leading to multiple testing issues [8]. An independent loop is defined as one that contains at least one edge not shared with any other independent loop [14]. The number of such independent loops is given by E−N+1 [15]. In our toy example in Figure 2, this results in 5−4+1=2 independent loops. However, there is no clear guideline on which loops should be selected for analysis.

#### 2.2.2 Side‐Splitting

Rather than focusing on closed loops of evidence, the side‐splitting method compares the different sources of evidence on each treatment comparison [13, 16]. Here, the direct evidence is compared to the pooled indirect estimate using the inconsistency factor, $\omega_{ij}$, where

(4) $$\omega_{ij}=|{\hat{\theta }}_{ij}^{\text{dir}}-{\hat{\theta }}_{ij}^{\text{indir(total)}}|.$$

Using this $\omega_{ij}$, a Z‐test is performed to assess the statistical significance of the inconsistency for the comparison i vs j [13]. By lumping together the indirect evidence, this approach can obscure inconsistencies between different indirect estimates. This is the case for the 1‐3 comparison in our toy example in Figure 2, where the pooled indirect evidence (see Equation (2)) equals the direct estimate (i.e, ${\hat{\theta }}_{ij}^{\text{dir}}=2$), resulting in no detected inconsistency ($\omega_{13}=0$, p−value=1). Here, the inconsistency between indirect estimates is masked when we focus on the 1‐3 comparison because the total indirect estimate equals the direct one. Conversely, the side‐splitting approach does detect inconsistency (p−value=0.006) for the treatment comparison 2 vs 3. The inconsistency in the network manifests in the results for this comparison.

#### 2.2.3 Design‐by‐Treatment Interaction

The previous two methods are described as local as they test for inconsistencies in particular areas of the network. The design‐by‐treatment interaction model instead calculates a global measure of inconsistency, which attempts to capture the inconsistency in the network as a whole. By introducing a parameter that captures differences between direct and indirect evidence, this method describes consistency and inconsistency models as multivariate meta‐regressions [8]. Using a Wald statistic test on the inconsistency parameter indicates a p‐value of 0.003 for the example in Figure 2. With a 95% significance level, this suggests that there is global inconsistency within the network. However, while the model successfully identifies the presence of inconsistency, it does not provide information about where the inconsistency is located.

#### 2.2.4 Net Heat Plot

Another method for detecting inconsistency is the net heat plot, a graphical representation of the extent of inconsistency associated with each design [7]. This method employs a Cochran‐like statistic, $Q^{\text{net heat}}$, [17] to quantify between‐design inconsistency and evaluates how this measure changes for a design d before and after another design $d^{\prime }$ is removed from the network, where the change is denoted as

$$\Delta Q_{d,d^{\prime }}^{\text{net heat}}=Q_{\text{before}}^{\text{net heat}}-Q_{\text{after}}^{\text{net heat}}.$$

Details of this calculation can be found in the original paper by Krahn et al. [7].

The resulting heatmap for our toy example can be obtained using the netheat() function in the netmeta package, [18] as shown in Figure 3. For simplicity, we consider only two‐arm trials, allowing the terms design and comparison to be used interchangeably. The gray squares have areas proportional to the contribution from the treatment comparison in the column to the treatment comparison in the row [7]. Each entry is color‐coded: positive values of $\Delta Q_{d,d^{\prime }}^{\text{net heat}}>0$ are shown in red, and negative values $\Delta Q_{d,d^{\prime }}^{\text{net heat}}<0$ are shown in blue.

**FIGURE 3:**
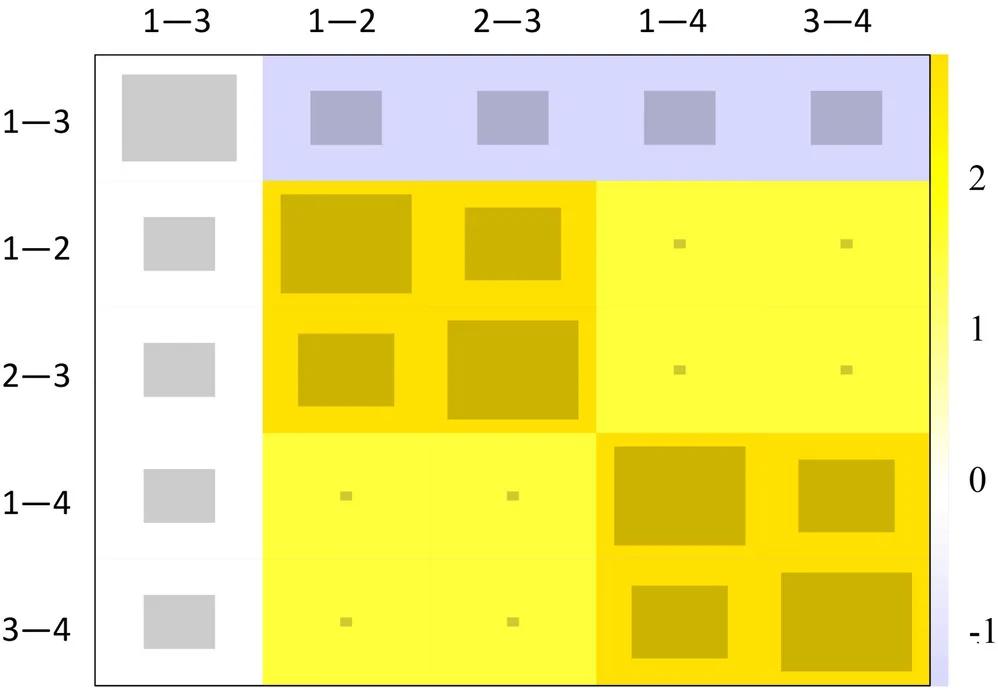
Net heat plot for the toy example in Figure 2. Each row and column represents a design; however, since only two‐arm trials are considered, they correspond to individual comparisons. The gray squares have areas proportional to the contribution from the treatment comparison in the column to the treatment comparison in the row. Blue indicates an increase of the inconsistency measure for the comparison in the row after the comparison in the column is removed from the network, while yellow and red indicate reduction of inconsistency.

The first row of the heatmap in Figure 3 represents changes in the inconsistency of comparison 1‐3 after each comparison in the column is removed. The colors in this row are all blue (and white when 1‐3 itself is detached), indicating negative values of $\Delta Q_{13,d^{\prime }}^{\text{net heat}}$. This suggests that removing any other comparison increases the inconsistency measure for 1‐3. However, as discussed in Section 2.1, there are three distinct sources of evidence for 1‐3, all of which are mutually inconsistent. Therefore, removing any comparison from the network in Figure 2 would be expected to reduce the measure of inconsistency, that is, $\Delta Q_{13,d^{\prime }}^{\text{net heat}}>0$. This observation indicates that the net heat plot does not account for the fact that the indirect evidence itself may be internally inconsistent.

## 3 Path‐Based Approach

In Section 2, we saw that the side‐splitting method and the net heat plot could not capture the conflicting evidence on the 1‐3 contrast in our toy example in Figure 2. The loop‐specific method, while more accurate, measures the inconsistency of an entire loop, and its implementation in large networks may prove to be resource‐intensive and lead to multiple testing. It also becomes less reliable when multi‐arm trials are present. On the other hand, the design‐by‐treatment interaction model is a global method, and does not provide a local analysis of the inconsistency. It also relies on modeling assumptions (for instance, the interaction term), and results may be sensitive to the chosen statistical model. If the model assumptions are not met, the results may be biased or misleading [8].

To address these limitations, we propose a new measure of inconsistency based on “paths of evidence” [5]. Previously, paths have been used to calculate the proportion contributions of direct comparisons in the context of NMA estimates [5, 15, 19]. Here, a path refers to a series of distinct nodes connected by direct comparisons. By using a path‐based approach, we ensure that we do not overlook conflicts that may arise from lumping all the indirect evidence. Instead, we consider each piece of indirect evidence individually. An additional benefit of this approach is that it does not require direct evidence to draw inferences regarding inconsistency between two treatments. As long as at least two independent sources of evidence are available, either one direct and one indirect source or two indirect sources, we can assess their consistency.

### 3.1 Hat Matrix and NMA Estimate

To elaborate on how the path‐based method works, we first have to understand how the NMA estimate is determined. Consider ${\hat{\theta }}^{\text{dir}}$ to be an M×1 vector containing all the summary effects for direct comparisons (${\hat{\theta }}_{ij}^{\text{dir}}$), with 0s for the comparisons that are not directly studied [15]. The variance associated with each direct estimate, $\sigma_{ij}^{2}$, is given by the inverse of the sum of inverse trial variances [11]. To handle the multi‐arm trials, we first adjust their variances using the method by Rücker et al. [10] to obtain a set of equivalent 2‐arm estimates. We calculate ${\hat{\theta }}_{ij}^{\text{nma}}$ for the NMA estimate of treatment comparison ij. We collect these estimates in the M×1 vector ${\hat{\theta }}^{\text{nma}}$ calculated via

$${\hat{\theta }}^{\text{nma}}=\text{H}{\hat{\theta }}^{\text{dir}},$$

where H is the M×M full hat matrix [15]. The hat matrix maps the vector of observed summary effects onto the vector of network estimates, and depends on the variances of the direct estimates and the structure of the network. Using the consistency assumption in Equation (1), each element ${\hat{\theta }}_{ij}^{\text{nma}}$ can then be expressed as a linear combination of direct estimates,

(5) $${\hat{\theta }}_{ij}^{\text{nma}}={\text{H}}_{ij,:}{\hat{\theta }}^{\text{dir}},$$

where ${\text{H}}_{ij,:}$ is the row of H corresponding to the comparison ij.

Geometrically, ${\hat{\theta }}^{\text{nma}}$ is the projection of ${\hat{\theta }}^{\text{dir}}$ onto an M‐dimensional subspace. For any row ${\text{H}}_{r,:}$, the element $h_{rc}$ indicates the leverage or influence that the column ${\text{H}}_{:,c}$ exerts on that row. Therefore, the leverages of the direct comparisons in the estimated NMA are indicated by the diagonal elements of the hat matrix, $h_{dd}$ [20]. For a specific comparison ij, one can obtain a directed network from node i to node j using the corresponding row ${\text{H}}_{r=ij,:}$. Every element $h_{rc}$ of this row represents an edge c=lk, and the direction of each edge is determined by the sign of $h_{rc}$ [5, 21]. That is, if $h_{rc}>0$, the edge points from node l to node k, while $h_{rc}<0$ corresponds to the direction k to l.

### 3.2 Path Inconsistency

Using the hat matrix, H, we start with the directed network for two treatments of interest, i and j. We illustrate two cases using the toy example in Figure 2: in the first case, the comparison of interest is 1‐3, and in the second, it is 2‐3. Figures 4(a) and 4(c) show the directed networks for these comparisons respectively. A path $\pi_{p}^{ij}$, is a series of distinct edges (or nodes) that starts at node i, passes through edges in the direction given by the sign of the corresponding entry in the ${\text{H}}_{ij,:}$ matrix row, and ends at node j. In another words, we can write a path as a set of distinct edges $\pi_{p}^{ij}=\{ik,\ldots ,k^{\prime }j\}$. The total number of paths between the pair of nodes i and j is denoted by P, with $\pi_{ij}$ representing the complete set of such paths, $\pi_{ij}=\{\pi_{1}^{ij},\ldots ,\pi_{P}^{ij}\}$. In this context, the direct comparison between i and j is just one path out of P paths between these two treatments, with only one edge in the path.

**FIGURE 4:**
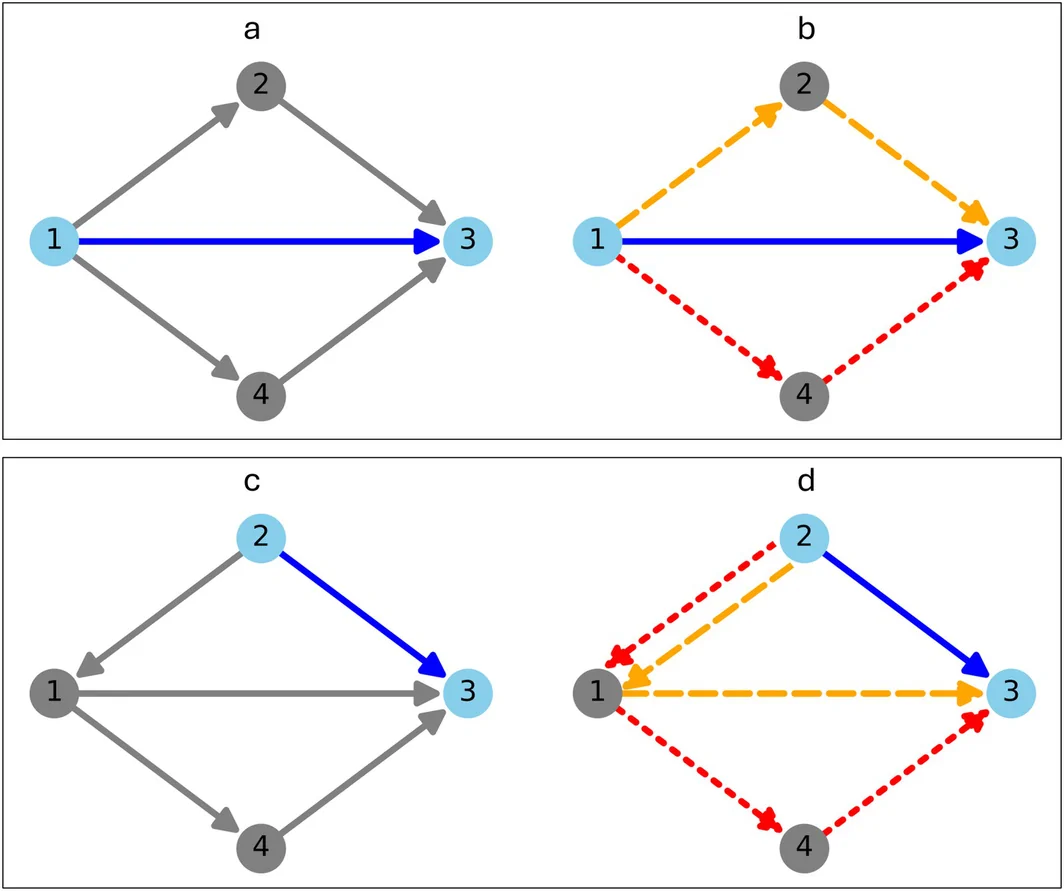
The directed network constructed from the ${\text{H}}_{ij,:}$ matrix row for (a) 1 vs 3, and (c) 2 vs 3. All paths of evidence (b) from 1 to 3, and (d) from 2 to 3. The direct path is shown in solid blue. Indirect paths are shown in dashed orange and dotted red.

Each path is associated with an effect, ${\hat{\theta }}_{ij}^{\pi_{p}}$, and a variance, $\mathrm{var}({\hat{\theta }}_{ij}^{\pi_{p}})$. The path effect is equal to the summation of the effects in the edges involved in that path,

(6) $${\hat{\theta }}_{ij}^{\pi_{p}}=\underset{kk^{\prime }\in \pi_{p}^{ij}}{\sum }{\hat{\theta }}_{kk^{\prime }}^{\text{dir}}.$$

Similarly, the path variance is the summation of the corresponding variances,

(7) $$\mathrm{var}({\hat{\theta }}_{ij}^{\pi_{p}})=\underset{kk^{\prime }\in \pi_{p}^{ij}}{\sum }\sigma_{kk^{\prime }}^{2}.$$

For each comparison of interest, we construct two vectors: (1) ${\hat{\theta }}_{ij}^{\pi }={\left({\hat{\theta }}_{ij}^{\pi_{1}},\ldots ,{\hat{\theta }}_{ij}^{\pi_{P}}\right)}^{⊤}$, a vector of length P containing the effect estimates of each path (Equation (6)), and (2) ${\hat{\theta }}_{ij}^{\text{nma}}={\hat{\theta }}_{ij}^{\text{nma}}I_{P\times 1}$, representing the network estimate for that comparison, where $I_{P\times 1}$ is a column vector of ones. We also define $\sum_{ij}$ as a P×P symmetric variance‐covariance matrix where each row and column represents a path. The diagonal elements of this matrix are the variances of each path (Equation (7)), and the off‐diagonal elements are the covariances between each pair of paths,

(8) $$\sum_{ij}=\left(\begin{array}{llll} \mathrm{var}({\hat{\theta }}_{ij}^{\pi_{1}}) & \text{cov}({\hat{\theta }}_{ij}^{\pi_{1}},{\hat{\theta }}_{ij}^{\pi_{2}}) & \ldots & \text{cov}({\hat{\theta }}_{ij}^{\pi_{1}},{\hat{\theta }}_{ij}^{\pi_{P}})\\ \text{cov}({\hat{\theta }}_{ij}^{\pi_{2}},{\hat{\theta }}_{ij}^{\pi_{1}}) & \mathrm{var}({\hat{\theta }}_{ij}^{\pi_{2}}) & \ldots & \text{cov}({\hat{\theta }}_{ij}^{\pi_{2}},{\hat{\theta }}_{ij}^{\pi_{P}})\\ \vdots & & & \\ \text{cov}({\hat{\theta }}_{ij}^{\pi_{P}},{\hat{\theta }}_{ij}^{\pi_{1}}) & \ldots & \ldots & \mathrm{var}({\hat{\theta }}_{ij}^{\pi_{P}}) \end{array}\right).$$

The covariance between two paths is the summation of the variances of all the edges that the two paths have in common.

For 1‐3 and 2‐3 in Figures 4(a) and 4(c), we detect three paths connecting the respective treatment pairs, as illustrated in Figures 4(b) and 4(d). For each comparison of interest, these paths are

(9) $$\begin{array}{ll} \begin{array}{c} ij=1‐3\\ \pi_{1}^{13}:1\to 3\\ \;\;\;\;\pi_{2}^{13}:1\to 2\to 3\\ \;\;\;\;\pi_{3}^{13}:1\to 4\to 3, \end{array} & \;\begin{array}{c} ij=2‐3\\ \pi_{1}^{23}:2\to 3\\ \;\;\;\;\;\pi_{2}^{23}:2\to 1\to 3\\ \;\;\;\;\;\;\pi_{3}^{23}:2\to 1\to 4\to 3. \end{array} \end{array}$$

Therefore for each case, the vectors of path and NMA estimates are

$$\begin{array}{ll} {\hat{\theta }}_{13}^{\pi } & =\left(\begin{array}{c} {\hat{\theta }}_{13}^{\pi_{1}}\\ {\hat{\theta }}_{13}^{\pi_{2}}\\ {\hat{\theta }}_{13}^{\pi_{3}} \end{array}\right)=\left(\begin{array}{c} 2\\ 1\\ 3 \end{array}\right),\;{\hat{\theta }}_{23}^{\pi }=\left(\begin{array}{c} {\hat{\theta }}_{23}^{\pi_{1}}\\ {\hat{\theta }}_{23}^{\pi_{2}}\\ {\hat{\theta }}_{23}^{\pi_{3}} \end{array}\right)=\left(\begin{array}{c} 0.5\\ 1.5\\ 2.5 \end{array}\right),\\ {\hat{\theta }}_{13}^{\text{nma}} & =\left(\begin{array}{c} {\hat{\theta }}_{13}^{\text{nma}}\\ {\hat{\theta }}_{13}^{\text{nma}}\\ {\hat{\theta }}_{13}^{\text{nma}} \end{array}\right)=\left(\begin{array}{c} 2\\ 2\\ 2 \end{array}\right),\;{\hat{\theta }}_{23}^{\text{nma}}=\left(\begin{array}{c} {\hat{\theta }}_{23}^{\text{nma}}\\ {\hat{\theta }}_{23}^{\text{nma}}\\ {\hat{\theta }}_{23}^{\text{nma}} \end{array}\right)=\left(\begin{array}{c} 1\\ 1\\ 1 \end{array}\right). \end{array}$$

Assuming all edges in our fictional example have the same variance ($0.3^{2}$), we find for ij=13

(10) $$\begin{array}{lll} \sum_{13} & =\left(\begin{array}{ccc} \sigma_{13}^{2} & 0 & 0\\ 0 & \sigma_{13}^{2}+\sigma_{23}^{2} & 0\\ 0 & 0 & \sigma_{13}^{2}+\sigma_{43}^{2} \end{array}\right)\; & =\left(\begin{array}{ccc} 0.3^{2} & 0 & 0\\ 0 & 2(0.3^{2}) & 0\\ 0 & 0 & 2(0.3^{2}) \end{array}\right), \end{array}$$

and for ij=23,

(11) $$\begin{array}{lll} \sum_{23} & =\left(\begin{array}{ccc} \sigma_{23}^{2} & 0 & 0\\ 0 & \sigma_{21}^{2}+\sigma_{13}^{2} & \sigma_{21}^{2}\\ 0 & \sigma_{21}^{2} & \sigma_{21}^{2}+\sigma_{14}^{2}+\sigma_{43}^{2} \end{array}\right)\; & =\left(\begin{array}{ccc} 0.3^{2} & 0 & 0\\ 0 & 2(0.3^{2}) & 0.3^{2}\\ 0 & 0.3^{2} & 3(0.3^{2}) \end{array}\right). \end{array}$$

When the comparison of interest is 2‐3, the paths $\pi_{2}^{23}$ and $\pi_{3}^{23}$ (the dashed orange and dotted red paths in Figure 4(d)) share one edge with each other, namely the comparison 1‐2. Therefore, the $\text{cov}({\hat{\theta }}_{23}^{\pi_{2}},{\hat{\theta }}_{23}^{\pi_{3}})$ in Equation (11) is equal to $\sigma_{12}^{2}$.

We define a statistic $Q_{ij}^{\text{path}}$ to measure the inconsistency between different paths of evidence for the comparison ij,

(12) $$Q_{ij}^{\text{path}}={\left({\hat{\theta }}_{ij}^{\pi }-{\hat{\theta }}_{ij}^{\text{nma}}\right)}^{⊤}{\left(\sum_{ij}\right)}^{-1}({\hat{\theta }}_{ij}^{\pi }-{\hat{\theta }}_{ij}^{\text{nma}}).$$

This measures, for comparison ij, how far away each path estimate is from the network estimate, weighted by the inverse path covariance matrix. In Figure 1, assuming additive treatment effects on a linear predictor scale, normally distributed effects for 1‐2 and 2‐3 comparisons imply normally distributed 1‐3 effects under the consistency assumption [22, 23]. Rücker et al. show that the network estimate for a pair of treatments is equal to a weighted mean of path estimates for that comparison [15]. In Equation (12), since ${\hat{\theta }}_{ij}^{\pi }$ and ${\hat{\theta }}_{ij}^{\text{nma}}$ are estimated from the same evidence, $Q_{ij}^{\text{path}}$ approximately follows a chi‐squared distribution $\chi_{P-1}^{2}$ with P−1 degrees of freedom (see Appendix A) [24, 25]. Therefore, we can use this measure to obtain a p‐value to test our null hypothesis of consistency, that is, that all paths of evidence connecting a given pair of treatments (whether direct or through any number of different indirect paths) yield the same estimate of the relative treatment effect.

In our toy example, we obtain a p‐value of 0.003 for both cases 1‐3 and 2‐3, which indicates significant inconsistency for these two comparisons.

### 3.3 Linearly Dependent Paths

The path inconsistency statistic in Equation (12) can only be evaluated if the path covariance matrix $\sum_{ij}$ is invertible. Since $\sum_{ij}$ is square, a lack of inverse indicates the matrix is singular. This is caused when some paths are linear combinations of other paths. To illustrate this, we introduce a second fictional example from Rücker et al. [15] shown in Figure 5. This network has N=5 treatments and E=7 direct comparisons. We focus on the comparison of 1 vs 3. Figure 5(b) shows the directed network of evidence obtained from the corresponding row of the H matrix. Due to a lack of direct evidence on this comparison, there is no direct path from 1 to 3. There are, however, five indirect paths labeled $\pi_{1}^{13}$, …, $\pi_{5}^{13}$ and shown in Figure 6.

**FIGURE 5:**
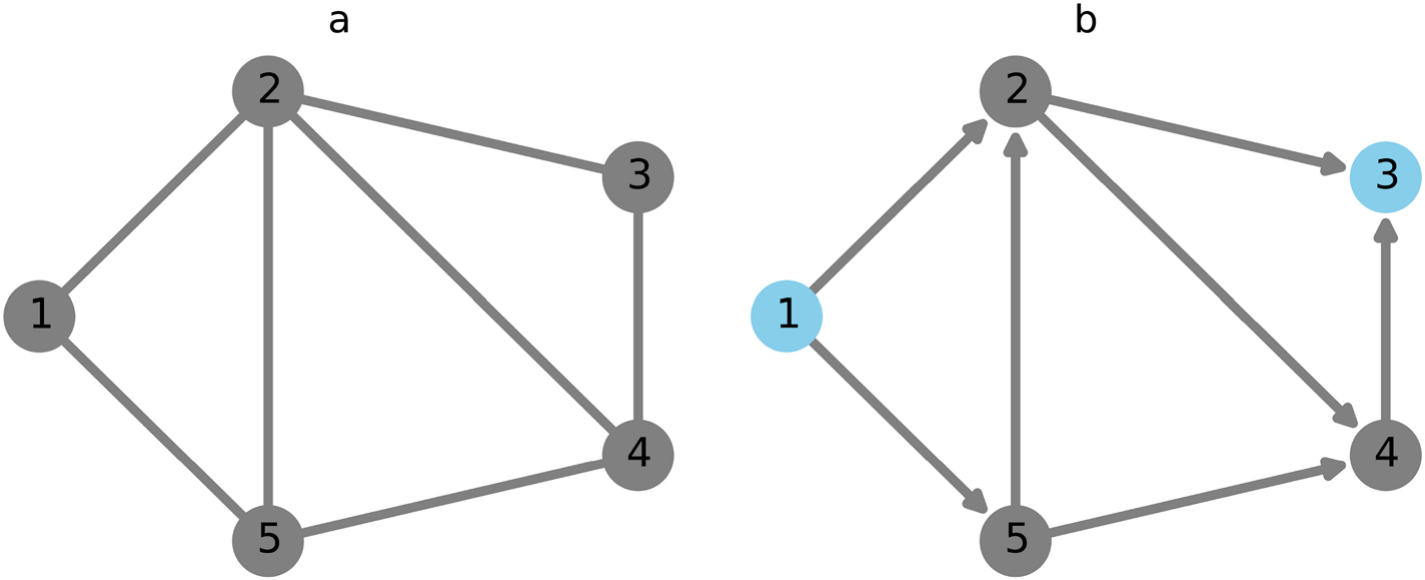
(a) Fictional example network from Rücker et al. [15] with five nodes and seven edges. (b) The directed network for the comparison 1 vs 3 constructed from the corresponding row of the H matrix.

**FIGURE 6:**
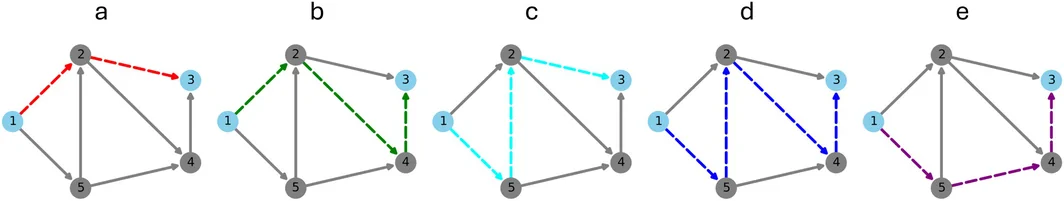
All the five paths of evidence between 1 and 3. (a) $\pi_{1}^{13}$, (b) $\pi_{2}^{13}$, (c) $\pi_{3}^{13}$, (d) $\pi_{4}^{13}$, and (e) $\pi_{5}^{13}$. [15]

We introduce the terms ‘adding’ and ‘subtracting’ paths to mean combining or removing all the edges involved in those paths. In Figure 7, we show that adding paths $\pi_{1}^{13}$ and $\pi_{4}^{13}$ gives the same set of directed edges as adding paths $\pi_{2}^{13}$ and $\pi_{3}^{13}$. Therefore, we can write the linear relation [15]

(13) $$\pi_{1}^{13}+\pi_{4}^{13}=\pi_{2}^{13}+\pi_{3}^{13}.$$

We call these paths “linearly dependent” [15] since any of the four paths $\pi_{1}^{13}$, $\pi_{2}^{13}$, $\pi_{3}^{13}$, and $\pi_{4}^{13}$ can be created by adding and subtracting the other three according to Equation (13). In general, a network may involve multiple linear relations between different subsets of paths, especially if the network is very large.

**FIGURE 7:**
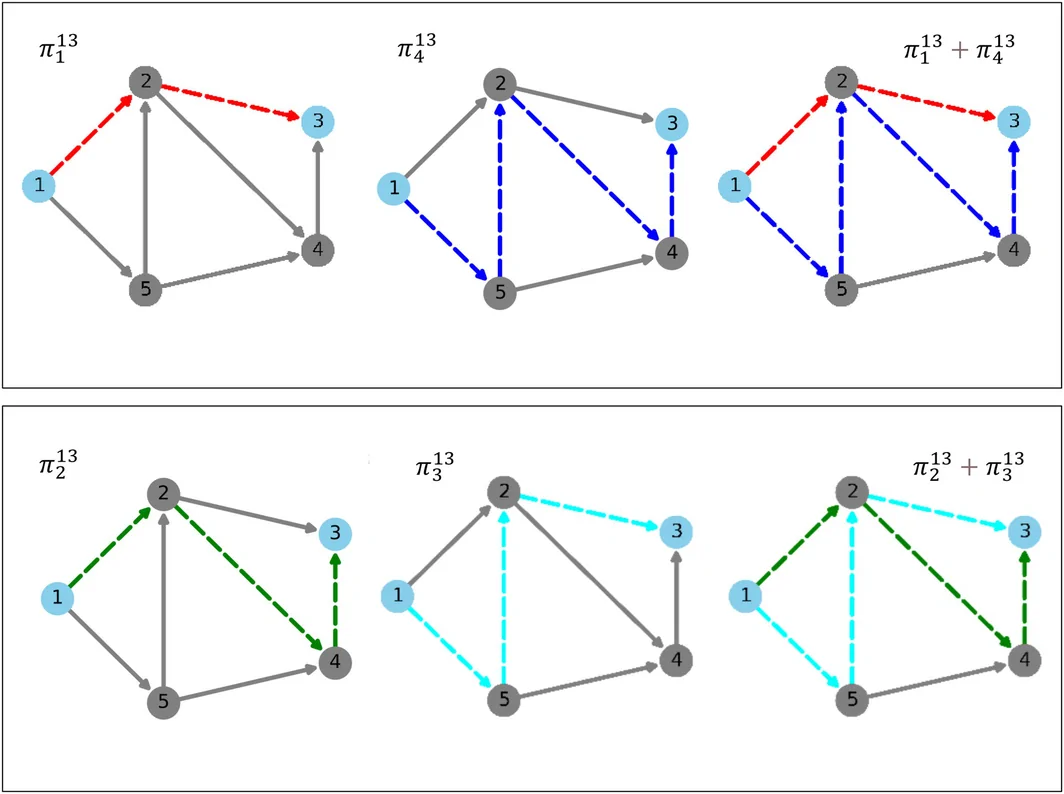
Depiction of the linear relation in Equation (13), where the left‐hand side, $\pi_{1}^{13}+\pi_{4}^{13}$, equals the right‐hand side, $\pi_{2}^{13}+\pi_{3}^{13}$.

In Section B1 of the Appendix we show that the covariance matrix $\sum_{ij}$ becomes invertible once all linear dependencies between paths are removed. Therefore, for each linearly dependent subset of paths in a network, we must break the dependency by removing the dependent paths. The choice of which path to remove does not affect the overall computation of $Q_{ij}^{\text{path}}$. Here, we can remove any of $\pi_{1}^{13}$, $\pi_{2}^{13}$, $\pi_{3}^{13}$, or $\pi_{4}^{13}$ from the $Q_{13}^{\text{path}}$ calculations, and still get the same results. This is because the respective path is removed from both the path estimate vector and the covariance matrix. Therefore, this choice is arbitrary. In Section B3 of the Appendix, we illustrate different choices of linearly independent path sets using a fictional example, and in Appendix B.1, we prove that $Q_{ij}^{\text{path}}$ is invariant to the choice of linearly independent path set. We then write $P^{\prime }<P$ for the number of independent paths remaining once the dependent paths have been removed, and these are the paths that we use in Equation (12).

To identify linearly dependent paths, we define ${\text{A}}_{ij}$ as a P×P symmetric path‐adjacency matrix, where each row and column represents a path. Diagonal elements, $a_{dd}$, contain the length of the corresponding path $\pi_{d}^{ij}$ and off‐diagonal elements, $a_{rc}$, are given by the number of shared edges between $\pi_{r}^{ij}$ and $\pi_{c}^{ij}$. ${\text{A}}_{ij}$ has full rank if and only if there is no dependency between paths. Therefore, we obtain a set of independent paths by reducing this matrix to its rank. For details, we refer to Sections B1 and B2 in the Appendix. Once the set of independent paths are identified, we define a reduced $P^{\prime }\times P^{\prime }$ path‐covariance matrix $\sum_{ij}$ via Equation (8) and a corresponding $P^{\prime }\times 1$ vector of path effects, ${\hat{\theta }}_{ij}^{\pi }$.

### 3.4 Netpath Plot

For a particular treatment comparison ij, the path inconsistency method measures how far away the network estimate of that comparison is from the estimate associated with each path of evidence. These deviations are aggregated into a single statistic $Q_{ij}^{\text{path}}$ (Equation (12)) which is used to test for the presence of inconsistency on that comparison. Following this assessment, one might then ask the following questions: (1) which paths contribute to the inconsistency, and (2) how large is the discrepancy between their estimates? To answer these questions, we propose a method for visualizing the extent of disagreement between pairs of paths. This takes our approach beyond just a test for inconsistency, allowing for a more granular exploration of where and to what extent inconsistencies occur.

For any pair of paths $p,p^{\prime }$, we define the absolute difference

$$d_{p,p^{\prime }}^{ij}\;=\;|{\hat{\theta }}_{ij}^{\pi_{p}}-{\hat{\theta }}_{ij}^{\pi_{p}^{\prime }}|.$$

The variance of this difference (accounting for covariances) is

$$\mathrm{var}(d_{p,p\prime }^{ij})\;=\;\mathrm{var}({\hat{\theta }}_{ij}^{\pi_{p}})+\mathrm{var}({\hat{\theta }}_{ij}^{\pi_{p}^{\prime }})-2\mathrm{cov}({\hat{\theta }}_{ij}^{\pi_{p}},{\hat{\theta }}_{ij}^{\pi_{p}^{\prime }}).$$

A natural standardized measure of distance is:

(14) $$z_{p,p^{\prime }}^{ij}\;=\;\frac{d_{p,p^{\prime }}^{ij}}{\sqrt{\mathrm{var}(d_{p,p^{\prime }}^{ij})}}.$$

Small $z_{p,p^{\prime }}^{ij}$ indicates the two estimates are close to each other, while large $z_{p,p^{\prime }}^{ij}$ indicates they are relatively far apart.

We plot these values as a heat map that shows the discrepancies between each pair of paths for a comparison ij. We call it the Netpath plot. Panels (a) and (b) in Figure 8 show this visualization for comparisons 1‐3 and 2‐3, respectively for our toy example in Figure 4. The darker colors indicate more conflict between the corresponding pair of paths. One could perform a statistical test based on these $\left|z_{p,p^{\prime }}^{ij}\right|$ values and plot the corresponding p‐values; however, we retain the absolute values for the representations here.

**FIGURE 8:**
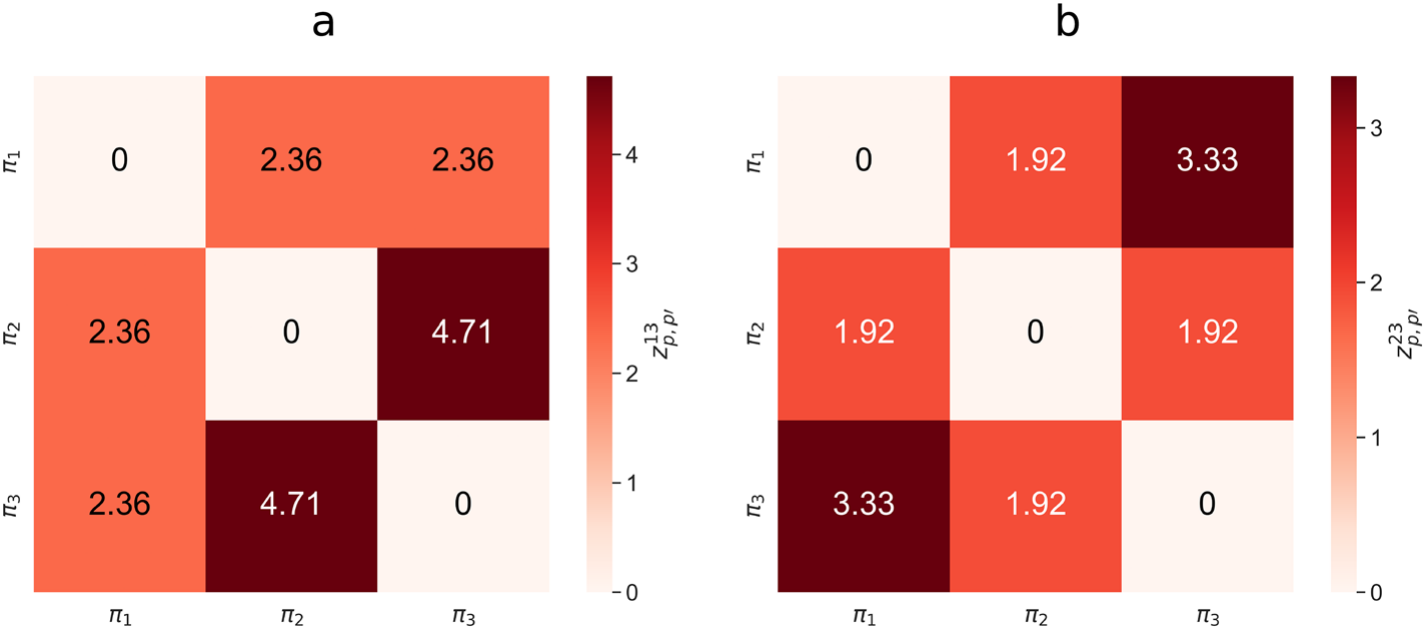
The Netpath plot to visualize Equation (14). Panel (a) corresponds to paths in Figure 4 (b); and panel (b) corresponds to paths in Figure 4 (d). These paths are also given in Equation (9). Darker shades of red correspond to greater disagreement between the two path estimates. As a result, all diagonal cells are white, reflecting the fact that $z_{p,p}^{ij}=0$. The superscripts in $\pi_{i}^{13}$ and $\pi_{i}^{23}$ are omitted for a less cluttered representation.

Figure 8(a) corresponds to our toy example in Figure 4(b) when the comparison of interest is 1‐3. The Netpath plot shows that the two indirect paths, $\pi_{2}^{13}$ (1→2→3) and $\pi_{3}^{13}$ (1→4→3), are equally inconsistent with the direct evidence $\pi_{1}^{13}$ (1→3). This means that the (absolute) difference between the estimates associated with paths $\pi_{1}^{13}$ and $\pi_{2}^{13}$ is the same as the (absolute) difference between paths $\pi_{1}^{13}$ and $\pi_{3}^{13}$. Indeed, this is the symmetry we specified in our set‐up that causes inconsistency to be masked when the indirect evidence is pooled.^1^ As expected, the highest degree of inconsistency on comparison 1‐3 is between the two indirect paths $\pi_{2}^{13}$ and $\pi_{3}^{13}$.

Figure 8(b) is the Netpath plot corresponding to our second case (comparison 2‐3) in Figure 4(d). This plot displays that the direct path $\pi_{1}^{23}$ (2→3) is in more conflict with $\pi_{3}^{23}$ (2→1→4→3) than it is with $\pi_{2}^{23}$ (2→1→3). This arises from the fact that the discrepancy between the direct path estimate (${\hat{\theta }}_{23}^{\pi_{1}}=0.5$) and the path estimate ${\hat{\theta }}_{23}^{\pi_{3}}=2.5$ is larger compared to the discrepancy with the path estimate ${\hat{\theta }}_{23}^{\pi_{2}}=1.5$.

Further insights are also possible. For example, we can calculate

(15) $$\underset{p^{\prime }}{\sum }z_{p,p^{\prime }}^{ij}$$

to obtain the row score of a specific path p. This corresponds to the summation of all scaled path‐effect differences for path p. In other words, it is the row summation of the Netpath plot entries. The path with the minimum row score represents the least disagreement with other paths, while the maximum row score indicates the greatest conflict. This row score ranks the paths between i and j according to the degree of disagreement in the evidence they provide for a given treatment comparison.

Figure 9 shows a bar heatmap to display these row scores for the two cases we investigated for our toy example in Figure 4. In Figure 9(a), the bar heatmap ranks the path evidence for the comparison between treatments 1 and 3 according to their row scores. We observe that $\pi_{1}^{13}$ shows the least disagreement with the other two paths, whereas $\pi_{2}^{13}$ and $\pi_{3}^{13}$ display the strongest and equal levels of disagreement. An intuitive explanation for this pattern lies in the symmetry of disagreement: $\pi_{1}^{13}$ (the direct path 1‐3) equally (symmetrically) conflicts with $\pi_{2}^{13}$ and $\pi_{3}^{13}$, while $\pi_{2}^{13}$ exhibits greater conflict with $\pi_{3}^{13}$ than it does with $\pi_{1}^{13}$. Figure 9(b) shows the bar heatmap that ranks the path evidence for the comparison between treatments 2 and 3 according to their row scores. Path $\pi_{2}^{23}$ shows the least disagreement with other paths overall.

**FIGURE 9:**
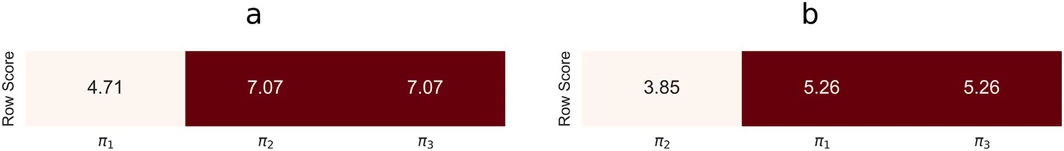
For each path, the row score in Equation (15) is calculated for: (a) Figure 8 (a), and (b) Figure 8 (b). These paths are also given in Equation (9). The row scores are sorted from the smallest (shown in lighter shades) to the largest (shown in darker shades) values.

To implement the path‐based inconsistency methods described here, we intend to add a new function netpath() to the netmeta package [26]. The function is currently available on GitHub [27]. It takes a netmeta object as its input, and outputs a summary of the path inconsistencies on any comparison of interest. This summary includes degrees of freedom, a p‐value for the null hypothesis test, and the Netpath plot. Additional detail is presented in the Supporting Information.

## 4 Real‐world example

### 4.1 Dataset

We apply our method to a network meta‐analysis in diabetes, comparing the effects of ten interventions on HbA1c values. These data were originally presented in Senn et al. [28] and have been preprocessed for use in the netmeta R package. We label the treatments as 1,…,10 (where 1= acarbose, 2= benfluorex, 3= metformin, 4= miglitol, 5= pioglitazone, 6= placebo, 7= rosiglitazone, 8= sitagliptin, 9= sulfonylurea, 10= vildagliptin). The dataset includes one three‐arm study and contains a total of 15 direct comparisons (edges). The corresponding network graph is shown in Figure 10(a).

**FIGURE 10:**
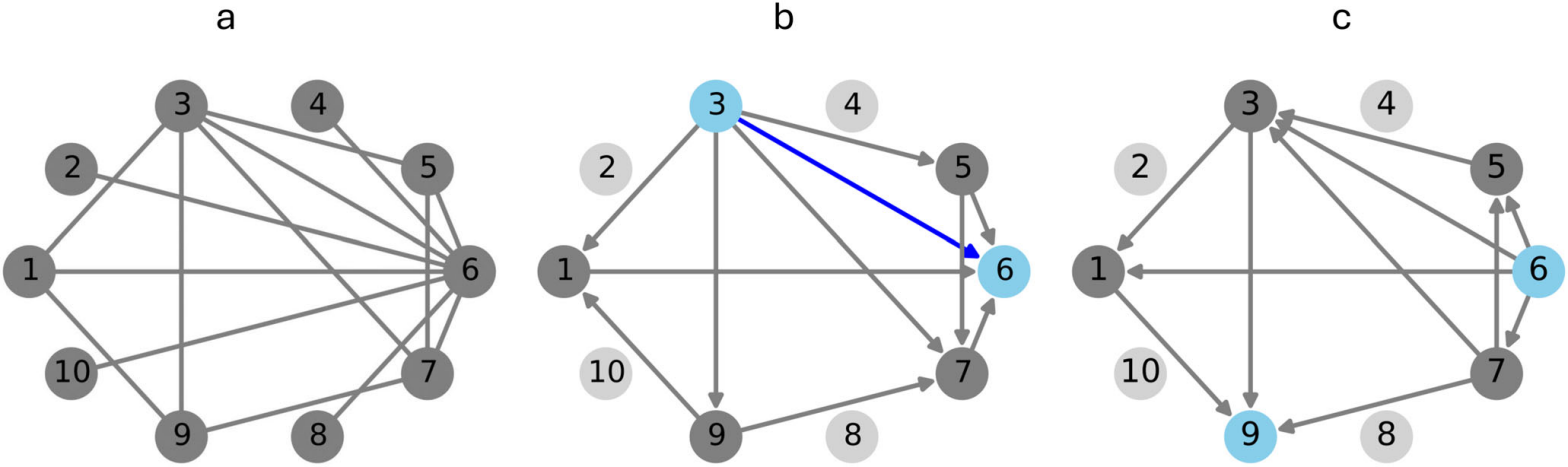
(a) The network graph for the NMA of diabetes with 10 interventions and 15 direct comparisons. [28] The corresponding directed networks for (b) comparison 3‐6 (metformin vs placebo), and (c) comparison 6‐9 (placebo vs sulfonylurea) constructed from the H matrix.

To demonstrate our approach, we consider two comparisons of interest: 3‐6 (metformin vs placebo) and 6‐9 (placebo vs sulfonylurea). For each of these comparisons, we compare our path‐based approach with the side‐splitting method, which is commonly used to assess inconsistency in network meta‐analysis. The loop‐specific approach becomes challenging in this network due to the presence of multi‐arm studies. Moreover, the network contains 35 loops, of which only 6 are independent; however, there are no clear guidelines for determining which loops should be considered independent. Therefore, we do not test the loop specific method on this example.

### 4.2 Metformin vs Placebo (Comparison With Direct Evidence)

We begin with treatment comparison 3 vs 6 (metformin vs placebo) which has been directly compared in four studies [28].

**Side splitting method:** The direct estimate of this comparison is equal to ${\hat{\theta }}_{36}^{\text{dir}}=-1.15$ with a variance of $\mathrm{var}({\hat{\theta }}_{36}^{\text{dir}})={\left(0.08\right)}^{2}$. The total indirect estimate is ${\hat{\theta }}_{36}^{\text{indir(total)}}=-1.06$ with a variance of $\mathrm{var}({\hat{\theta }}_{36}^{\text{indir(total)}})={\left(0.09\right)}^{2}$. Running a Z‐test, this results in a p‐value of 0.45 indicating no significant inconsistencies at a 95% significance level. As in our toy example, this can be attributed to the agreement between the total indirect evidence and the direct evidence within their confidence intervals. As we will see below, the inconsistencies among the indirect sources of evidence are not detected.

**Path‐based method:** We start by calculating the H matrix using the hatmatrix() function in the netmeta package. Then, we construct a directed network of evidence for these two chosen treatments based on the 3‐6 row in the H matrix. The corresponding row vector, expressed in terms of treatment labels {1,…,10}, is given by:

$$\begin{array}{l} \begin{array}{cc} & (1‐2,\;1‐3,\;\ldots ,\;2‐3,\;2‐4,\;\ldots ,\;3‐4,\;\;\text{……}\;\;\text{……}\\ & \;\;\text{……}\;\;\text{……}\;\;\text{……}\;,9‐10)\\ H_{36,:}= & (0,\;-2,\;0,\;0,\;5,\;0,\;0,\;-4,\;0,\;\ldots 0,\;18,\;58,\;11,\;0,\;11,\;0,\;\;\\ & \;\ldots \;0,\;14,\;5,\;0,\;0,\;0,\;-23,\;0,\;\ldots \;0,\;-7,\;0,\;\ldots \;0)\times 0.01. \end{array} \end{array}$$

Here, the dots in the index row indicate continuation over all pairwise treatment comparisons, while the dots in the numeric row indicate consecutive zero entries. This directed network is shown in Figure 10(b). When an entry in the $H_{ij,:}$ is zero, it means that the corresponding column does not provide any information about the comparison of interest ij. Figure 10(b) illustrates that the treatments 2,4,8, and 10 do not provide any information on the comparison 3‐6.

We then apply a depth‐first search method [29] to this directed network, uncovering a total of P=7 distinct paths from 3 to 6. These paths are shown in Table 1.

**TABLE 1:** Paths and their estimates for treatment comparison 3 (metformin) vs 6 (placebo).

Path label	Path sequence	Path sequence with clinical names	${\hat{\theta }}_{36}^{\pi_{i}}$	$\mathrm{var}({\hat{\theta }}_{36}^{\pi_{i}})$
$\pi_{1}^{36}$	{3→1→6}	metformin → acarbose → placebo	−1.02	0.15
$\pi_{2}^{36}$	{3→6}	metformin → placebo	−1.15	0.006
$\pi_{3}^{36}$	{3→9→1→6}	metformin → sulfonylurea → acarbose → placebo	−0.79	0.06
$\pi_{4}^{36}$	{3→9→7→6}	metformin → sulfonylurea → rosiglitazone → placebo	−0.32	0.04
$\pi_{5}^{36}$	{3→5→6}	metformin → pioglitazone → placebo	−1.46	0.02
$\pi_{6}^{36}$	{3→5→7→6}	metformin → pioglitazone → rosiglitazone → placebo	−1.21	0.04
$\pi_{7}^{36}$	{3→7→6}	metformin → rosiglitazone → placebo	−1.07	0.03

Consequently, the path‐adjacency matrix for this comparison, $A_{36}$, has dimensions 7×7 and it is equal to

(16) $${\text{A}}_{36}=\left(\begin{array}{ccccccc} 2 & 0 & 1 & 0 & 0 & 0 & 0\\ 0 & 1 & 0 & 0 & 0 & 0 & 0\\ 1 & 0 & 3 & 1 & 0 & 0 & 0\\ 0 & 0 & 1 & 3 & 0 & 1 & 1\\ 0 & 0 & 0 & 0 & 2 & 1 & 0\\ 0 & 0 & 0 & 1 & 1 & 3 & 1\\ 0 & 0 & 0 & 1 & 0 & 1 & 2 \end{array}\right).$$

${\text{A}}_{36}$ is a full rank matrix. This means all the paths between 3 and 6 are linearly independent of one another.

Now, we construct the vector of path effect estimates based on Table 1 as

(17) $${\hat{\theta }}_{36}^{\pi }={\left(-1.02,-1.15,-0.79,-0.32,-1.46,-1.21,-1.07\right)}^{⊤}.$$

Substituting this vector into Equation (5) (e.g., using the netmeta package), and assuming a common‐effect model, yields a network estimate of ${\hat{\theta }}_{36}^{\text{nma}}=-1.11$. The vector of network estimates is then constructed as ${\hat{\theta }}_{36}^{\text{nma}}={\hat{\theta }}_{36}^{\text{nma}}I_{7\times 1}$. The variance‐covariance matrix is calculated as

$$\sum_{36}=\left(\begin{array}{ccccccc} 0.15 & 0 & 0.02 & 0 & 0 & 0 & 0\\ 0 & 0.01 & 0 & 0 & 0 & 0 & 0\\ 0.02 & 0 & 0.06 & 0.01 & 0 & 0 & 0\\ 0 & 0 & 0.01 & 0.04 & 0 & 0.003 & 0.003\\ 0 & 0 & 0 & 0 & 0.02 & 0.01 & 0\\ 0 & 0 & 0 & 0.003 & 0.01 & 0.04 & 0.003\\ 0 & 0 & 0 & 0.003 & 0 & 0.003 & 0.03 \end{array}\right).$$

Finally, inserting ${\hat{\theta }}_{36}^{\pi }$, ${\hat{\theta }}_{36}^{\text{nma}}$, and $\sum_{36}$ into Equation (12) yields $Q_{36}^{\text{path}}=22.45$. Based on the assumption of a $\chi_{7-1}^{2}$ distribution with 6 degrees of freedom, this gives a p‐value of 0.001, which, at the 95% significance level, suggests that there is significant evidence for inconsistency. This result reflects the variation in the path estimates in Equation (17), which range from −0.32 to −1.15. This was not captured by the side‐splitting method because pooling the indirect estimates gives an estimate (−1.06) that is similar to the direct estimate (−1.15).

Going even further, we can use the path approach to investigate the sources of this inconsistency using the Netpath plot shown in Figure 11. This reveals that, among all pairs of paths, the greatest conflict is between the estimates associated with paths $\pi_{4}^{36}$ and $\pi_{5}^{36}$ ($z_{4,5}^{36}=4.64$). These two paths are shown in Figure 12(a). At the other extreme, paths $\pi_{1}^{36}$ and $\pi_{7}^{36}$ show the least disagreement, with the minimum value of $z_{1,7}^{36}=0.14$. These two paths are shown in Figure 12(b).

**FIGURE 11:**
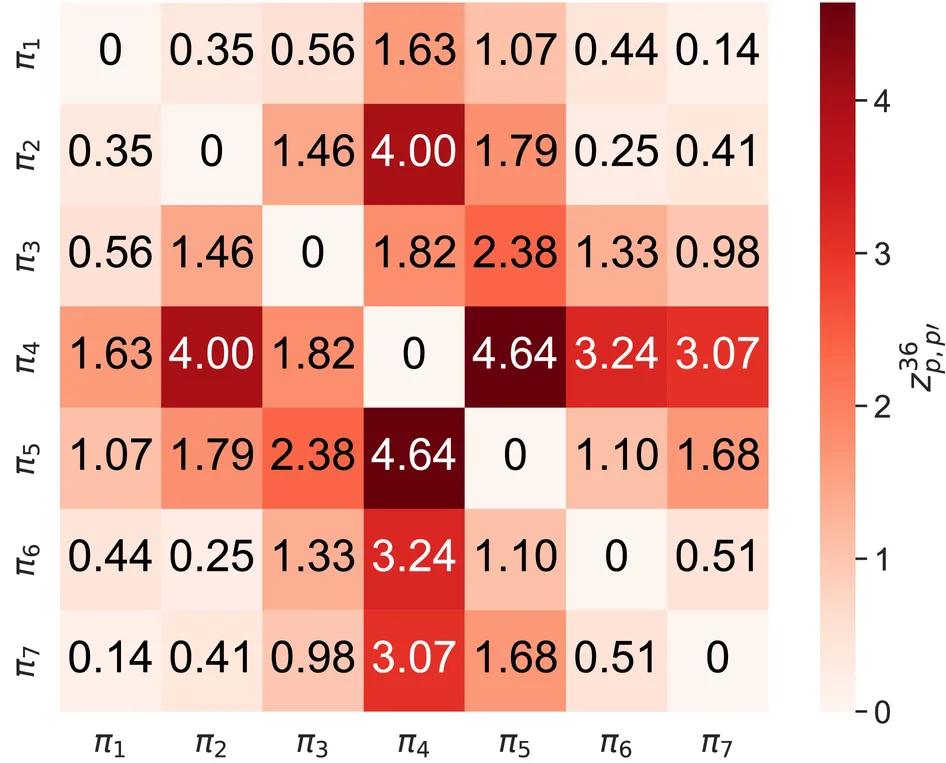
Inconsistency Netpath plot for all the independent paths between 3 and 6 in Figure 10 (b). Darker shades of red correspond to greater disagreement between the two path estimates. As a result, all diagonal cells are white, reflecting the fact that $z_{p,p}^{36}=0$. The superscript in $\pi_{p}^{36}$ is omitted for clarity.

**FIGURE 12:**
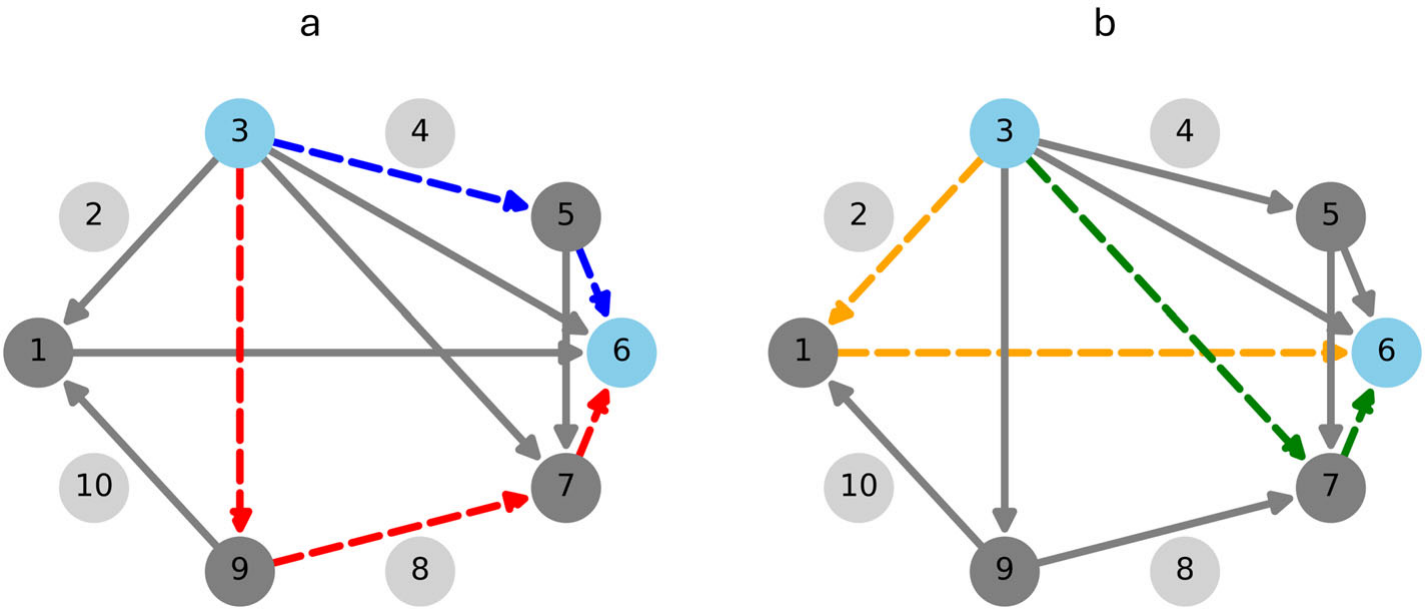
(a) Paths $\pi_{4}^{36}$ (red dashed line) and $\pi_{5}^{36}$ (blue dashed line) exhibit the greatest disagreement with each other. (b) Paths $\pi_{1}^{36}$ (orange dashed line) and $\pi_{7}^{36}$ (green dashed line) exhibit the least disagreement with each other.

Using the row score, we can then further identify the path primarily responsible for the observed inconsistency. In Figure 13, we order the paths of evidence for 3 vs. 6 according to their row scores. The path with the minimum row score represents the least disagreement with other paths, while the maximum row score indicates the greatest conflict. Figure 13 shows a bar heatmap to display these row scores. We observe that path $\pi_{4}^{36}$, via treatments 9 (sulfonylurea) and 7 (rosiglitazone), has the strongest disagreement with the other paths overall, whereas path $\pi_{1}^{36}$, via treatment 1 (acarbose), is the most consistent.

**FIGURE 13:**
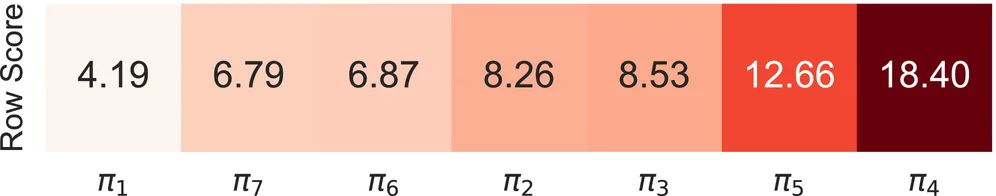
The row score (see Equation (15)) of every path between 3 and 6. Path $\pi_{1}^{36}$ shows the least (shown in light pink) while path $\pi_{4}^{36}$ shows the most conflict (shown in dark red) with other paths. Here, $\pi_{2}^{36}$ is a direct path from 3 (metformin) to 6 (placebo). The paths and their estimates can be obtained from Table 1.

### 4.3 Placebo vs Sulfonylurea (Comparison Without Direct Evidence)

We now focus on the comparison between treatment 6 (placebo) and treatment 9 (sulfonylurea). As shown in Figure 10(a), there is no trial comparing these two treatments directly. Consequently, the side‐splitting and loop‐specific methods cannot be applied, and the net heat plot cannot assess any inconsistency around this comparison. In contrast, the path‐based method allows us to investigate inconsistency for this comparison by examining the agreement between different paths. A direct comparison can be viewed as just one possible path, and its absence does not prevent the assessment of inconsistency, provided that at least two independent sources of evidence (paths) are available for the comparison.

**Path‐based method:** To perform our path‐based analysis on the comparison 6‐9, we construct the directed network shown in Figure 10(c) from the corresponding row in the hat matrix, ${\text{H}}_{69,:}$. This row is given in Section **B** of the Supporting Information. Applying the depth‐first search algorithm [29] yields a total of P=10 paths from node 6 to node 9, shown in Table 2 with their corresponding estimates. Not all of these paths are linearly independent. Paths $\pi_{5}^{69},\pi_{7}^{69}$, and $\pi_{10}^{69}$ create linearly dependent rows in our path‐adjacency matrix, ${\text{A}}_{69}$. Therefore, we reduce the ${\left({\text{A}}_{69}\right)}_{10\times 10}$ matrix to its rank of 7, resulting in $P^{\prime }=7$ independent paths between 6 and 9. Since the paths between 6‐9 cover the same set of comparisons as those between 3‐6, the corresponding inconsistency measure is identical, that is, $Q_{69}^{\text{path}}=Q_{36}^{\text{path}}=22.45$. With six degrees of freedom, this results in a p‐value of 0.001.

**TABLE 2:** Paths and their estimates for treatment comparison 6 (placebo) vs 9 (sulfonylurea). The clinical names of treatments are abbreviated here.

Path label	Path sequence	Path sequence with clinical names	${\hat{\theta }}_{69}^{\pi_{i}}$	$\mathrm{var}({\hat{\theta }}_{69}^{\pi_{i}})$
$\pi_{1}^{69}$	{6→3→1→9}	pla → met → aca → sul	0.55	0.16
$\pi_{2}^{69}$	{6→3→9}	pla → met → sul	0.78	0.02
$\pi_{3}^{69}$	{6→1→9}	pla → aca → sul	0.42	0.04
$\pi_{4}^{69}$	{6→5→3→1→9}	pla → pio → met → aca → sul	0.86	0.17
$\pi_{5}^{69}$	{6→5→3→9}	pla → pio → met → sul	1.09	0.04
$\pi_{6}^{69}$	{6→7→3→1→9}	pla → ros → met → aca → sul	0.47	0.18
$\pi_{7}^{69}$	{6→7→3→9}	pla → ros → met → sul	0.70	0.04
$\pi_{8}^{69}$	{6→7→9}	pla → ros → sul	−0.05	0.02
$\pi_{9}^{69}$	{6→7→5→3→1→9}	pla → ros → pio → met → aca → sul	0.61	0.19
$\pi_{10}^{69}$	{6→7→5→3→9}	pla → ros → pio → met → sul	0.84	0.06

Furthermore, although no direct comparison is available for this case, we can still examine the agreement among different indirect sources of evidence using the Netpath and row score plots shown in Figures 14 and 15. Figure 14 shows that the largest conflict exists between path $\pi_{2}^{69}$ (through treatment 3 (metformin)) and path $\pi_{8}^{69}$ (through treatment 7 (rosiglitazone)). Figure 15 shows that path $\pi_{9}^{69}$ (via 7 (rosiglitazone), 5 (pioglitazone), 3 (metformin), and 1 (acarbose)), shows the least disagreement with other paths with a row score of 4.11; while path $\pi_{8}^{69}$ (via 7 (rosiglitazone)), shows the most conflict with other paths with a row score of 11.9. This may be due to the fact that rosiglitazone is the most efficacious treatment in this network, such that paths through this node tend to produce results different from other paths.

**FIGURE 14:**
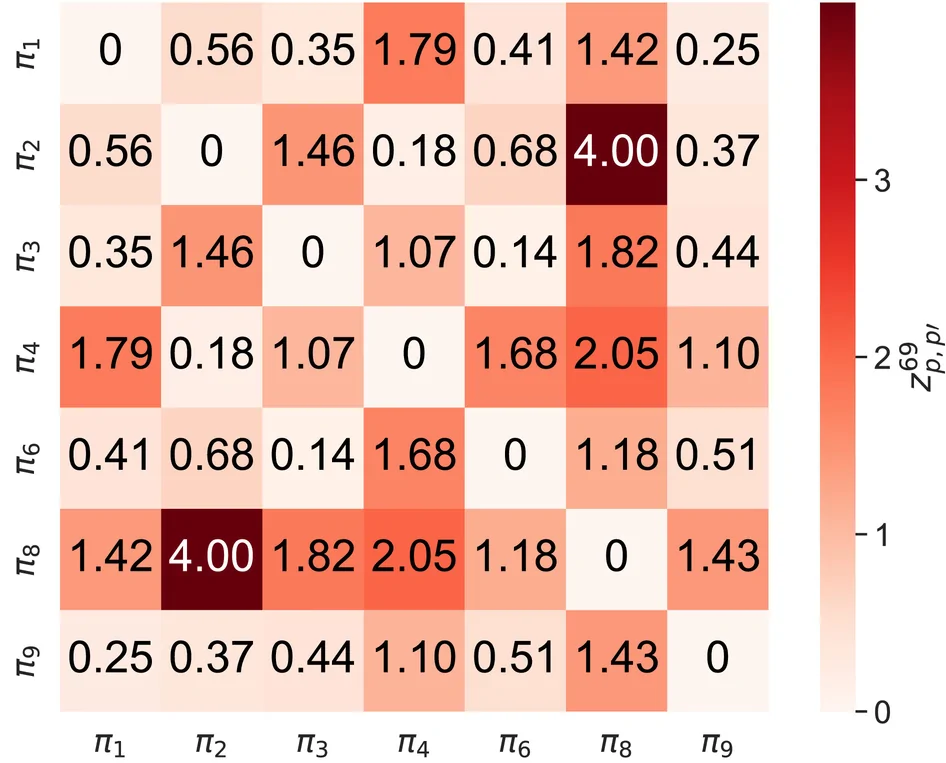
Inconsistency Netpath plot for all the independent paths between treatment 6 (placebo) and treatment 9 (sulfonylurea) in Figure 10 (c). Darker shades of red correspond to greater disagreement between the two path estimates. As a result, all diagonal cells are white, reflecting the fact that $z_{p,p}^{69}=0$. The superscript in $\pi_{i}^{69}$ is omitted for clarity.

**FIGURE 15:**
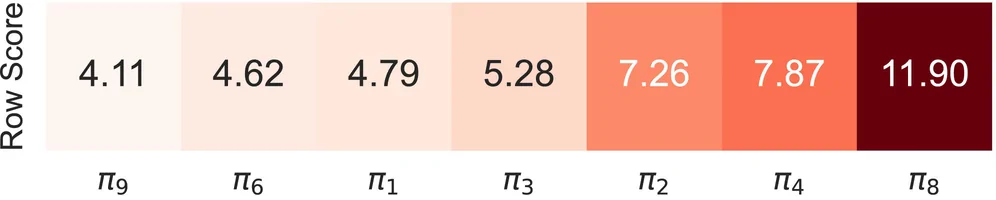
The row score (see Equation (15)) of every linearly independent path between 6 and 9. Path $\pi_{9}^{69}$ shows the least (shown in light pink) while path $\pi_{8}^{69}$ shows the most conflict (shown in dark red) with other paths. Here, none of the paths are a direct path between the two treatments 6 (placebo) and 9 (sulfonylurea). The paths and their estimates can be obtained from Table 2. The superscript in $\pi_{i}^{69}$ is omitted for clarity.

## 5 Discussion

Network meta‐analysis (NMA) has become increasingly valuable for addressing complex clinical questions involving multiple treatments, as it enables decision‐makers to evaluate more than two interventions simultaneously. However, the validity of NMA results depends on the consistency assumption as it pools all sources of evidence (direct and indirect) to estimate the efficacy of a treatment comparison. This makes robust methods for inconsistency assessment essential for reliable inference.

Existing approaches to inconsistency assessment, while widely used, exhibit important limitations that can compromise their conclusions. The loop‐specific approach, which focuses on closed loops within the network, does not have a clear way of handling dependent loops and encounters difficulties when dealing with multi‐arm trials. The side‐splitting method, which separates direct from indirect evidence for each comparison, can mask inconsistencies by aggregating all indirect evidence into a single estimate. This happens when the total indirect evidence happens to align with the direct evidence, even when individual indirect paths show substantial disagreement with each other. This aggregation obscures important conflicts between different sources of indirect evidence that may have clinical or methodological significance. The net heat plot is a visualization tool designed to display the contribution of direct evidence to network estimates and identify potential inconsistency hotspots. However, as demonstrated in our toy examples, one can obtain misleading interpretations in certain network configurations, potentially causing researchers to overlook genuine inconsistencies. Finally, a measure similar to ours was previously proposed by Caldwell et al. [30] but this was limited to comparisons where direct evidence is available and any indirect paths do not overlap (i.e, have no shared edges). In fact, all these established methods face challenges when direct evidence is unavailable for a comparison of interest.

To address these limitations, we introduce a path‐based approach that offers an alternative way to evaluate inconsistency in NMA. We introduce a quantitative measure of inconsistency that aims to identify discrepancies between different indirect paths of evidence. Importantly, this assesses each piece of evidence separately rather than aggregating all indirect evidence, and thereby avoids the masking effects sometimes observed in traditional methods. Furthermore, our approach does not rely on the presence of direct evidence. This enables a thorough investigation of inconsistency for comparisons where the direct evidence is absent, provided at least two indirect sources of evidence are available. Finally, we go beyond merely testing for inconsistency; we illustrate the extent of inconsistencies between different sources of evidence using a Netpath plot. This provides a local insight, focusing on one treatment comparison at a time (based on the user's clinical or methodological interest) and comparing all the different sources of evidence on that comparison.

Although the number of possible paths between two treatments can increase rapidly in networks with many treatments, this does not lead to an unmanageable output. The depth‐first search algorithm efficiently enumerates all possible paths, and the path‐adjacency matrix is then reduced to its independent set (see Appendix B2). Consequently, users work only with the independent paths, resulting in a manageable set even in large networks. In practice, the implementation in the netpath() function is computationally efficient, as illustrated by the accompanying R script in the Supporting Information. As an example, the analysis of the diabetes data in Section 4 is performed almost instantaneously. Computation time is expected to increase slightly only for substantially larger networks or when a large number of treatment comparisons are evaluated.

We have illustrated the path‐based approach through two toy examples and one empirical dataset, demonstrating its applicability to networks of varying complexity. These examples show how the framework, together with the proposed inconsistency measure and Netpath plot visualization, can help identify and characterize inconsistencies within a network. We recommend pre‐specifying comparisons of interest in the protocol stage, based on clinical and methodological judgment, and then reporting

- A p‐value for an approximate chi‐squared test for inconsistency in the network.
- A Netpath plot for each pre‐specified comparison that visualizes the relative disagreement between different sources of evidence. The plot has dimension $P^{\prime }\times P^{\prime }$, where $P^{\prime }$ denotes the number of independent paths (typically much smaller than the total number of possible paths).
- A row score plot (Figures 13 and 15) that shows which paths contribute most to the inconsistency on each pre‐specified comparison.

We recommend avoiding relying solely on the *p*‐values. The primary limitation of the path‐based approach is that it is based on a statistical test and therefore relies on p‐values to assess significance. Consequently, it inherits the well‐known limitations of hypothesis testing and p‐value‐based inference [31]. Additionally, the $\chi^{2}$ test proposed in Equation 12 is only an approximation; the finite‐sample calibration of this approximate test would benefit from further empirical or simulation‐based evaluation.

The path‐based approach thus complements existing methods by offering both an overall assessment of the network and detailed insights into individual comparisons. We note, that although $Q_{ij}^{\text{path}}$ is calculated for a comparison ij, it provides a global measure of inconsistency for the network. More specifically, when the set of paths between two treatments, i and j, cover the same comparisons as the set of paths between two other treatments, $i^{\prime }$ and $j^{\prime }$, the values of $Q_{ij}^{\text{path}}$ and $Q_{i^{\prime }j^{\prime }}^{\text{path}}$ are equal. However, if there are two subgraphs in the network that are connected only via a bridge (a single comparison or a single treatment), then each subgraph has its own global $Q_{ij}^{\text{path}}$. Local insights, on the other hand, are obtained via the Netpath and row score plots.

Although the Netpath plot and row score provide local insights into the sources of disagreement within the network, identifying the underlying causes of inconsistency requires further investigation. Inconsistency is closely linked to the transitivity assumption, which remains a fundamental yet conceptually complex requirement in NMA. Transitivity implies that if treatments are comparable through an intermediate common comparator, then indirect comparisons are valid. Ensuring transitivity in practice is challenging due to potential differences in study characteristics, patient populations, intervention implementations, or other effect modifiers across studies. When transitivity is violated, it can manifest as inconsistency within the network, but discerning the root cause of such inconsistency is not always straightforward. Observed inconsistencies may arise from genuine violations of transitivity, model misspecifications, or random variability. The current ambiguity in the relationship between transitivity and consistency limits our ability to interpret and address transitivity violations fully. Therefore, while our method offers a valuable tool for exploring and quantifying inconsistency, careful attention to the transitivity assumption, and consideration of clinical and methodological heterogeneity across studies, remains crucial for drawing reliable conclusions from NMA.

## Funding

This work was supported by the Deutsche Forschungsgemeinschaft (Grant Nos. NI 2226/1‐1 and Project‐ID 499552394 SFB 1597) and the Engineering and Physical Sciences Research Council (Grant No. EP/Y007905/1).

## Disclosure

Authors Noosheen R. Tahmasebi, Theodoros Papakonstantinou, and Adriani Nikolakopoulou were supported by the Deutsche Forschungsgemeinschaft (DFG, German Research Foundation) ‐ grant number NI 2226/1‐1. Adriani Nikolakopoulou has received funding from the European Research Executive Agency under the HORIZON TMA MSCA Postdoctoral Fellowships ‐ European Fellowships grant agreement 101244226. Author Annabel L. Davies received funding from the Engineering and Physical Sciences Research Council (EPSRC) EP/Y007905/1.

## Conflicts of Interest

The authors declar no conflicts of interest.

## Supporting information



supinfo/sim70752‐sup‐0001‐Supinfo.zip.

## Figures and Tables

**FIGURE B1:**
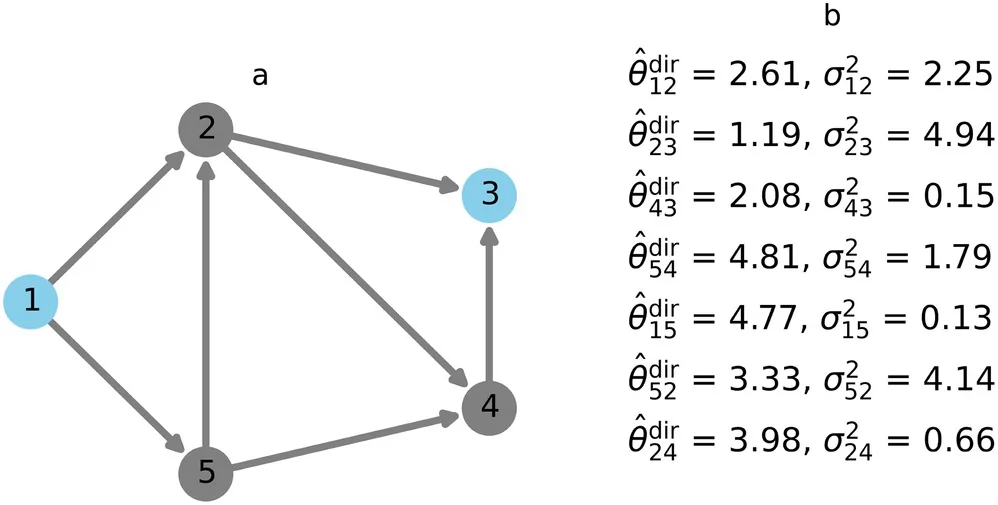
(a) Fictional example in Figure 5 with (b) arbitrary values for the effects and variances for the seven direct comparisons.

## Data Availability

Data sharing not applicable to this article as no datasets were generated or analysed during the current study.

## ENDNOTES

^1^
Note that this masking effect can still occur even without symmetry; we could have increased the discrepancy of one path and decreased the discrepancy on the other as long as their average was still equal to the direct estimate.

## Appendix A Degrees of Freedom for a Chi‐Squared Distribution

For a normally distributed n‐dimensional vector $x∼N_{n}(\mu ,\sum )$, with true mean μ and covariance matrix ∑, the expression

(A1)
$${\left(x-\mu \right)}^{T}{\left(\sum \right)}^{-1}(x-\mu )$$

follows a chi‐squared distribution $\chi_{n}^{2}$ with n degrees of freedom [32]. This, however, applies when μ is the true population mean, a known fixed parameter that does not depend on the particular realization of x; that is, it is exogenous to the draw. Cochran [33] shows that the residual quadratic form from a k‐parameter fit to an n‐dimensional Gaussian loses exactly k degrees of freedom, giving a $\chi_{n-k}^{2}$ distribution. This indicates that if $\mu =\bar{x}$ is a scalar mean estimated from the entries of the vector x, one degree of freedom (k=1) is consumed because μ is estimated from the data using the same metric in which the expression in Equation A1 is measured. In other words, the true mean is unknown, and we estimate an expected value using the sample mean, $\bar{x}$ [34].

## Appendix B Linearly Independent Paths

### B1 Singularity of the Covariance Matrix

In this part, we prove that by removing the linearly dependent paths, the covariance matrix $\sum_{ij}$ will be always invertible unless there is a direct comparison with a variance of 0.

We define the path‐edge incidence matrix ${\text{C}}_{ij}$ as a P×E matrix where each row represents a path between nodes i and j, and each column represents an edge (direct comparison). In this matrix, an element is 1 if the corresponding edge is part of the path, and 0 otherwise. The path‐adjacency matrix, ${\text{A}}_{ij}$, can then be written as

(A2)
$${\text{A}}_{ij}={\text{C}}_{ij}{\left({\text{C}}_{ij}\right)}^{⊤}.$$

Using the path‐edge incidence matrix, we can also define the variance‐covariance matrix as

(A3)
$$\sum_{ij}={\text{C}}_{ij}\text{V}{\left({\text{C}}_{ij}\right)}^{⊤},$$

where V is an E×E diagonal matrix containing edge variances, with diagonal elements $v_{dd}$ representing the variance of comparison d.

The determinant of a diagonal matrix is the product of its diagonal elements, which means $\det(V)=\overset{E}{\underset{d=1}{\prod }}v_{dd}$. Therefore, according to Equation B2, $\sum_{ij}$ becomes singular when the variance of at least one direct comparison, $v_{dd}$, is 0, when $C_{ij}$ is rank‐deficient, or both. This indicates that if all the variances of direct comparisons are strictly positive, $\det\;\left(\sum_{ij}\right)\neq 0$ if and only if $\text{rank}(C_{ij})=P$.

Since V has strictly positive diagonal entries, it admits a well‐defined square root $V^{1/2}=\text{diag}\;\left(\sqrt{v_{11}},\ldots ,\sqrt{v_{EE}}\right)$, and we may write

$$\sum_{ij}=C_{ij}\;V^{1/2}V^{1/2}{\left(C_{ij}\right)}^{⊤}=M\;M^{⊤},\;\text{where}\;M=C_{ij}V^{1/2}.$$

The matrix $\sum_{ij}=M\;M^{⊤}$ is P×P and positive semi‐definite, hence it is invertible, if and only if M has full row rank, that is, rank(M)=P. Since $V^{1/2}$ is diagonal with strictly positive entries it is invertible, and therefore

$$\text{rank}(M)=\text{rank}\;\left(C_{ij}V^{1/2}\right)=\text{rank}(C_{ij}).$$

Combining these observations,

$$\begin{array}{ll} & \det\left(\sum_{ij}\right)\neq 0\;⟺\;\sum_{ij}\;\text{is positive definite}\;⟺\;\text{rank}(\text{M})=P\\ & \;\;⟺\;\text{rank}({\text{C}}_{ij})=P. \end{array}$$

According to Equation (B1), $\text{rank}(A_{ij})=\text{rank}(C_{ij})$. Therefore, removing dependent paths ensures that the matrix $A_{ij}$ becomes full‐rank, which guarantees a non‐zero determinant for $A_{ij}$ and, consequently, for $\sum_{ij}$.^2^

The path‐edge incidence matrix for the simple network structure in the second case of our first toy example in Figure 4b is

where each column represents 2−3,2−1,1−3,1−4, and 4−3 respectively. For this example, we considered the same variance for all the comparisons ($\sigma_{ij}^{2}=\sigma^{2}=0.3^{2}$); Therefore, the edge variance matrix is

$$\begin{array}{l} \text{V}={\left(\begin{array}{ccccc} \sigma_{23}^{2} & 0 & 0 & 0 & 0\\ 0 & \sigma_{21}^{2} & 0 & 0 & 0\\ 0 & 0 & \sigma_{13}^{2} & 0 & 0\\ 0 & 0 & 0 & \sigma_{14}^{2} & 0\\ 0 & 0 & 0 & 0 & \sigma_{43}^{2} \end{array}\right)}_{5\times 5}={\left(\begin{array}{ccccc} \sigma^{2} & 0 & 0 & 0 & 0\\ 0 & \sigma^{2} & 0 & 0 & 0\\ 0 & 0 & \sigma^{2} & 0 & 0\\ 0 & 0 & 0 & \sigma^{2} & 0\\ 0 & 0 & 0 & 0 & \sigma^{2} \end{array}\right)}_{5\times 5}. \end{array}$$

Entering this **V** matrix and $C_{23}$ into Equation (B2), we obtain the invertible variance‐covariance matrix shown in Equation (11).

### B2 Reducing a Rank‐Deficient Matrix to Its Rank

A square matrix ^3^ is called to be rank‐deficient if its rows (or columns) are linearly dependent. In another words, the dimension of the matrix is greater than its rank. In this section, we use our second toy example in Figure 5 to explain how one can reduce a rank‐deficient matrix and obtain a full rank matrix.

For the two treatments 1 and 3 in the toy example in Figure 5, five paths are detected via the depth‐first search [29]. These paths are shown in Figure 6. We create the path‐adjacency matrix, $A_{13}$ as

The diagonal elements in $A_{ij}$ represent the number of edges in that path. The off‐diagonal elements represent the number of edges that the two paths share. $A_{ij}$ matrix is the product of the $ZZ^{⊤}$, where Z is the path‐design matrix in Rücker et al. [15].

To reduce a rank‐deficient matrix to its rank, we employ the Row Echelon Form (REF) method, [35] which is favored for its efficiency and numerical stability, particularly useful in handling large matrices and computational tasks. The method identifies linearly dependent rows which are removed along with their equivalent columns to create a square full rank matrix. The algorithms that perform the Echelon Form method can be found in the literature, such as those described by Lay [36]. Variations of this method are also available in many functions in R and Python including the rref() function from Python package sympy, [37] or fullrank() function from R package robustbase, [38] or even qr() function from the base R [39]. We use all of these functions to ensure that the following step‐by‐step illustration is not dependent on any specific programming function.

Denoting every row in Matrix B.3 as $r_{i}$, the following operations are done step by step to transform $A_{13}$ to ${\tilde{A}}_{13}$ in B4:

$$\begin{array}{ll} \text{steps} & \;\text{operation}\\ s_{1} & \;r_{2}=-\frac{1}{2}r_{1}+r_{2}\\ s_{2} & \;r_{3}=-\frac{1}{2}r_{1}+r_{3}\\ s_{3} & \;r_{3}=\frac{1}{5}r_{2}+r_{3}\\ s_{4} & \;r_{4}=-\frac{4}{5}r_{2}+r_{4}\\ s_{5} & \;r_{5}=-\frac{2}{5}r_{2}+r_{5}\\ s_{6} & \;r_{4}=-r_{3}+r_{4}\\ s_{7} & \;r_{5}=-\frac{1}{2}r_{3}+r_{5}, \end{array}$$

(A4)
$${\tilde{A}}_{13}={\left(\begin{array}{ccccc} 2 & 1 & 1 & 0 & 0\\ 0 & \frac{5}{2} & -\frac{1}{2} & 2 & 1\\ 0 & 0 & \frac{12}{5} & \frac{12}{5} & \frac{6}{5}\\ 0 & 0 & 0 & 0 & 0\\ 0 & 0 & 0 & 0 & 2 \end{array}\right)}_{5\times 5}.$$

To create the REF, one must have the row(s) of zeroes at the bottom of the matrix (to get the so‐called echelon shape). Therefore, the echelon form of $A_{13}$ will be one step further (step $s_{8}$) by swapping the fourth with the fifth row. This yields

(A5)
$$\text{REF}(A_{13})={\left(\begin{array}{ccccc} 2 & 1 & 1 & 0 & 0\\ 0 & \frac{5}{2} & -\frac{1}{2} & 2 & 1\\ 0 & 0 & \frac{12}{5} & \frac{12}{5} & \frac{6}{5}\\ 0 & 0 & 0 & 0 & 2\\ 0 & 0 & 0 & 0 & 0 \end{array}\right)}_{5\times 5}.$$

A full row of zeroes in the row echelon form in B5 represents a deficiency in rank by one dimension for our path‐adjacency matrix, $A_{13}$. We keep the track of the indices of the rows that are swapped. Looking at ${\tilde{A}}_{13}$ in B4, we observe that the fourth row is a full row of 0s, which means the fourth path does not provide distinctive information. This indicates that the fourth row (path) can be expressed as a linear combination of other rows (paths). Therefore, removing this row and the corresponding fourth column does not diminish any information provided by the matrix. By removing the fourth row and column, now, we have a 4×4 full‐rank matrix as

According to the reduced $A_{13}$ in (B.6), we now have $P^{\prime }=4$ linearly independent paths. Therefore, we write the variance‐covariance matrix for this example as

(A6)
$$\sum_{13}={\left(\begin{array}{cccc} \sigma_{12}^{2}+\sigma_{23}^{2} & \sigma_{12}^{2} & \sigma_{23}^{2} & 0\\ \sigma_{12}^{2} & \sigma_{12}^{2}+\sigma_{24}^{2}+\sigma_{43}^{2} & 0 & \sigma_{43}^{2}\\ \sigma_{23}^{2} & 0 & \sigma_{15}^{2}+\sigma_{52}^{2}+\sigma_{23}^{2} & \sigma_{15}^{2}\\ 0 & \sigma_{43}^{2} & \sigma_{15}^{2} & \sigma_{15}^{2}+\sigma_{54}^{2}+\sigma_{43}^{2} \end{array}\right)}_{4\times 4}.$$

### B3 Different Sets of Linearly Independent Paths

The REF of a matrix, if it exists, is not unique. A rank‐deficient matrix can have multiple echelon forms depending on its linearly dependent rows. In $A_{13}$ in Equation (B.3), $r_{1}$, $r_{2}$, $r_{3}$, and $r_{4}$ are linearly dependent. Therefore, there are sequences of steps and operations that result in an ${\tilde{A}}_{13}$ with a full row of zeroes appearing in the first row, the second row, or the third row. This implies that any of the rows $r_{1}$, $r_{2}$, $r_{3}$, or $r_{4}$, along with their corresponding columns, can be removed to obtain a full‐rank matrix. This reflects the concept described in Equation (13), which allowed us to remove any of the paths $\pi_{1}^{13}$, $\pi_{2}^{13}$, $\pi_{3}^{13}$, or $\pi_{4}^{13}$ to form a set of linearly independent paths. This indicates that different full‐rank variance‐covariance matrices can be generated depending on the specific row(s) removed. We use Gaussian elimination to perform row operations that transform the initial matrix into one of the following matrices:

(A7)
$$\begin{array}{ccc} {\left({\tilde{A}}_{13}\right)}_{1} & =\left(\begin{array}{cccccc} & 0 & 0 & 0 & 0 & 0\\ & 0 & 2.5 & -0.5 & 2 & 1\\ & 0 & 2.5 & -0.5 & 2 & 1\\ & 0 & 2 & 2 & 4 & 2\\ & 0 & 1 & 1 & 2 & 3 \end{array}\right),\;{\left({\tilde{A}}_{13}\right)}_{2} & =\left(\begin{array}{cccccc} & \frac{5}{3} & 0 & 1 & \frac{-2}{3} & \frac{-1}{3}\\ & 0 & 0 & 0 & 0 & 0\\ & 1 & 0 & 3 & 2 & 1\\ & \frac{-2}{3} & 0 & 2 & \frac{8}{3} & \frac{4}{3}\\ & \frac{-1}{3} & 0 & 1 & \frac{4}{3} & \frac{8}{3} \end{array}\right),\\ {\left({\tilde{A}}_{13}\right)}_{3} & =\left(\begin{array}{cccccc} & \frac{5}{3} & 1 & 0 & \frac{-2}{3} & \frac{-1}{3}\\ & 1 & 3 & 0 & 2 & 1\\ & 0 & 0 & 0 & 0 & 0\\ & \frac{-2}{3} & 2 & 0 & \frac{8}{3} & \frac{4}{3}\\ & \frac{-1}{3} & 1 & 0 & \frac{4}{3} & \frac{8}{3} \end{array}\right),\;{\left({\tilde{A}}_{13}\right)}_{4} & =\left(\begin{array}{cccccc} & 2 & 1 & 1 & 0 & 0\\ & 1 & 2 & -1 & 0 & 0\\ & 1 & -1 & 2 & 0 & 0\\ & 0 & 0 & 0 & 0 & 0\\ & 0 & 0 & 0 & 0 & 2 \end{array}\right). \end{array}$$

Each of these matrices corresponds to a specific REF.

An important observation is that no matter which row is removed, the value of $Q_{ij}^{\text{path}}$ remains unchanged. This is because as the row and the corresponding column are removed from the variance‐covariance matrix, the respective row is also removed from the path and NMA estimate vectors. This means that the choice of linearly independent paths does not affect the results or inferences related to the measure of inconsistency. This is analytically proven below in Appendix B.1.

An illustrative toy example for this is given below in Figure B1, where we consider the arbitrary values for the effects and variances of the direct comparisons.

Here, the comparison of interest is 1‐3, where four out of five paths are linearly dependent according to Equation (13). Extensive but easy calculations show that the $Q_{13}^{\text{path}}$ is the same if any of these four paths is removed from the calculations. This means that it does not matter which path ($\pi_{1}^{13}$, $\pi_{2}^{13}$, $\pi_{3}^{13}$, or $\pi_{4}^{13}$) is removed, the chosen set of linearly independent paths will give us the same result, with $Q_{13}^{\text{path}}=3.32$. Since the DoF is 4, it will result in the same p‐value for the inconsistency of 1 vs 3 (p−value=0.5).

### B.1 Invariance of $Q_{ij}^{\text{path}}$ Under Different Sets of Independent Paths

In the following, we provide an analytical proof of the claim made in Appendix B3 that the statistic $Q_{ij}^{\text{path}}$ remains invariant with respect to the choice of linearly independent paths. To avoid cluttered super‐ and subscripts, we use $Q^{\text{path}}$ to refer to $Q_{ij}^{\text{path}}$, ∑ to refer to $\sum_{ij}$, and vector v to refer to $\left({\hat{\theta }}_{ij}^{\pi }-{\hat{\theta }}_{ij}^{\text{nma}}\right)$. Therefore, $Q^{\text{path}}$ in Equation 12 is rewritten as

(A8)
$$Q^{\text{path}}=v^{⊤}\sum^{-1}v.$$

Generally, v is a P×1 vector, and ∑ is a symmetric P×P matrix. If ∑ is rank‐deficient, then $P^{\prime }<P$, where $P^{\prime }$ denotes its rank. In this case, the inverse of ∑ does not exist, and therefore we have to approximate it via the Moore‐Penrose pseudoinverse [40]. $Q^{\text{path}}$ then becomes

(A9)
$$Q^{\text{path}}=v^{⊤}\sum^{+}v.$$

$\tilde{\sum }$ is a $P^{\prime }\times P^{\prime }$ full rank matrix constructed by reducing the rank‐deficient matrix, ∑ to its rank. From Appendix B2, we know that $\tilde{\sum }$ is not unique. $\left\{{\tilde{\sum }}_{1},{\tilde{\sum }}_{2},\ldots ,{\tilde{\sum }}_{r},\ldots \;\right\}$ represents the set of all possible full rank matrices. Respectively, $\left\{{\tilde{v}}_{1},{\tilde{v}}_{2},\ldots ,{\tilde{v}}_{r},\ldots \;\right\}$ are the reduced v vectors; recall that when we reduce ∑ to its rank, we remove the corresponding entries from v as well. Equation B10 is then written as

(A10)
$${\tilde{Q}}_{r}^{\text{path}}={\tilde{v}}_{r}^{⊤}{\tilde{\sum }}_{r}^{-1}{\tilde{v}}_{r},$$

where the inverse of ${\tilde{\sum }}_{r}$ exists. Equation B11 creates a set of $\left\{{\tilde{Q}}_{1}^{\text{path}},{\tilde{Q}}_{2}^{\text{path}},\ldots ,{\tilde{Q}}_{r}^{\text{path}},\ldots \;\right\}$, where we are creating a $Q^{\text{path}}$ measure for each and every possible reduced matrix, ${\tilde{\sum }}_{r}$. In Appendix B3, we claim $Q^{\text{path}}$ is invariant under different sets of independent paths. This means

(A11)
$${\tilde{Q}}_{1}^{\text{path}}\;=\;{\tilde{Q}}_{2}^{\text{path}}\;=\;\ldots \;=\;{\tilde{Q}}_{r}^{\text{path}}\;=\;\ldots .$$

To prove the statement in Equation B12, we start by the eigendecomposition of a matrix to express a rank‐deficient matrix, ∑, as

(A12)
$$\sum =U\;\Lambda \;U^{⊤},$$

where Λ is a diagonal $P^{\prime }\times P^{\prime }$ matrix that contains the eigenvalues of ∑, and U is a $P\times P^{\prime }$ unitary matrix ($U^{⊤}U=I_{P^{\prime }}$) [41]. We define $R_{r}$ to be a selection matrix picking out one of the ${\tilde{\sum }}_{r}$ and $\tilde{v_{r}}$ from their set, by removing rows, columns or entries from ∑ and v. As a result, we can write

(A13)
$$\begin{array}{ll} {\tilde{\sum }}_{r} & =R_{r}\;\sum \;R_{r}^{⊤}, \end{array}$$

(A14)
$$\begin{array}{ll} {\tilde{v}}_{r} & =R_{r}v. \end{array}$$

Using Equation B13, we write ${\tilde{\sum }}_{r}$ in Equation B14 in terms of the eigen values:

(A15)
$$\begin{array}{cc} {\tilde{\sum }}_{r} & =R_{r}\;(U\;\Lambda \;U^{⊤})\;R_{r}^{⊤}\\ & =K_{r}\;\Lambda \;K_{r}^{⊤}, \end{array}$$

where we define $K_{r}=R_{r}\;U$. $K_{r}$ is a full rank $P^{\prime }\times P^{\prime }$ matrix.

Since each path is a series of edges, we write the vector v ^4^ in terms of the path‐edge incidence matrix, $C_{ij}$:

$$\begin{array}{cc} v & =({\hat{\theta }}_{ij}^{\pi }-{\hat{\theta }}_{ij}^{\text{nma}})\\ & =C_{ij}{\hat{\theta }}^{\text{dir}}, \end{array}$$

where ${\hat{\theta }}^{\text{dir}}$ contains the edge effects. Looking at the Equation B2, one can deduce that v∈Range(∑), and since Range(∑)⊆Range(U), one can write v=Ub, where b is a $P^{\prime }\times 1$ coordinate vector that is expressed in the orthonormal eigen basis (U) that spans Range(∑). Combined with Equation B15, we write

(A16)
$${\tilde{v}}_{r}\;=\;K_{r}\;b.$$

We use Equations (A14), (A15), and B17 to expand ${\tilde{Q}}_{r}^{\text{path}}$ in Equation B11:

(A17)
$$\begin{array}{cc} {\tilde{Q}}_{r}^{\text{path}}\; & =\;{\tilde{v}}_{r}^{⊤}{\left(K_{r}\;\Lambda \;K_{r}^{⊤}\right)}^{-1}\;{\tilde{v}}_{r}\\ & =\;b^{⊤}\;K_{r}^{⊤}\;{\left(K_{r}^{⊤}\right)}^{-1}\;\Lambda^{-1}\;K_{r}^{-1}\;K_{r}\;b\\ & =\;b^{⊤}\;\Lambda^{-1}\;b, \end{array}$$

where we used the fact that matrices $K_{r}$ and Λ satisfy the necessary and sufficient conditions [42] such that ${\left(K_{r}\;\Lambda \;K_{r}^{⊤}\right)}^{-1}\;=\;{\left(K_{r}^{⊤}\right)}^{-1}\;\Lambda^{-1}\;K_{r}^{-1}$. Equation B18 indicates that the ${\tilde{Q}}_{r}^{\text{path}}$ measure defined in Equation B11 is independent of the chosen matrices from the set of $\left\{\tilde{\sum_{r}}\right\}$ and $\left\{\tilde{v_{r}}\right\}$. In other words, this proves the statement in Equation B12.

It is noteworthy that when the rows of a square matrix are linear combinations of other rows, it indicates that the matrix has more rows than the dimension of its row space, which means we are defining the space with more rows than needed. This does not affect the norm of a vector in this space. If we have a transformation matrix, T, the norm of a vector $\vec{x}$ is defined as ${\vec{x}}^{⊤}T\vec{x}$. This implies that $Q_{ij}^{\text{path}}$, representing the norm of the effect deviations $\left({\hat{\theta }}_{ij}^{\pi }-{\hat{\theta }}_{ij}^{\text{nma}}\right)$, remains unchanged whether all paths or only the linearly independent paths (the basis vectors in our path space) are used. Below, we provide an analytical proof to show that $Q^{\text{path}}$ is equal to the Equation B12.

We use v=Ub to expand $Q^{\text{path}}$ in Equation B10:

(A18)
$$\begin{array}{cc} Q^{\text{path}}\; & =\;b^{⊤}U^{⊤}\;\sum^{+}\;\text{Ub}\\ & =\;b^{⊤}\;\Lambda^{-1}\;b, \end{array}$$

where we used the eigendecomposition of the covariance matrix in Equation B13 to write $U^{⊤}\;\sum^{+}\;U=\Lambda^{-1}$. The $Q^{\text{path}}$ in Equation B19 is the same as the ${\tilde{Q}}_{r}^{\text{path}}$ in Equation B18. This indicates that the measure in Equation 12 is invariant under different sets of paths.

This implies that we can technically use the rank‐deficient ${\left(A_{ij}\right)}_{P\times P}$ in Equation  (B.3) and still obtain the same value for our measure of inconsistency, $Q_{ij}^{\text{path}}$. For the example discussed in this Appendix B3, we find $Q_{13}^{\text{path}}=3.32$, even without removing any row or column from $\sum_{13}$. This demonstrates that the same value for $Q_{ij}^{\text{path}}$ can be obtained using all paths of evidence between two treatments, i and j without removing any dependency. However, there are two important considerations to keep in mind:

1. Understanding the number of linearly independent paths remains essential for determining the degrees of freedom. This degree of freedom is crucial for performing a statistical test and obtaining a p‐value for the null hypothesis; and
2. when using all paths, the variance‐covariance matrix is no longer invertible ($\det(\sum_{ij})=0$). Consequently, a pseudo‐inverse method must be employed to approximate ${\left(\sum_{ij}\right)}^{-1}$ [40, 43].
