# Supplementary material for: A Path‐Based Approach for Detecting and Assessing Inconsistency in Network Meta‐Analysis
Source: Stat Med. 2026 Oct 15;45(23-24):e70752. doi: 10.1002/sim.70752 (PMC13672679; doi:10.1002/sim.70752)
Supplement: Supplementary file 1 [file SIM-45-0-s001.zip › Spporting_information.pdf]

Supporting Information

A | SOFTWARE

We intend to implement our path-based method in the `netmeta` package in `R`. Currently, it is accessible through the `netpath()` function via Github (`https://github.com/guido-s/netmeta/tree/netpath`). One can download and use the function through the following command:

```
remotes::install_github("guido-s/netmeta", ref = "develop")
```

First, we use the `netmeta` package in `R` to call `netmeta()`. This creates a `netmeta` object, `nma_obj`, which we then use in `netpath()` and `netheat()`. For a specific comparison of interest, the function `heatplot.netpath(nma_obj)` displays a Netpath plot like Figure 8 in the main text. We use `netpath(nma_obj, node1, node2)` to perform a depth-first search algorithm and obtain a full list of P paths, along with the respective statistics on their size, total effect, and variance. It also prints Q_{ij}^{path}, p-value, and the number of independent paths:

```
netpath_result
#> ---------- Path-based inconsistency test ----------
#> Comparison: 1 vs 3
#> Number of independent paths: 3
#> Quadratic form Q: 11.11
#> Degrees of freedom: 2
#> p-value: 0.003
#> ---------------------------------------------------
```

B | HAT MATRIX

The following Table shows the full 45×45 hat matrix for the diabetes network in Figure 10 in the main text. The name of the treatments are abbreviated as follows:
aca: acarbose, ben: benfluorex, met: metformin, mig: miglitol, pio: pioglitazone, pla: placebo, ros: rosiglitazone, sit: sitagliptin, sul: sulfonylurea, vil: vildagliptin.
Columns that consist entirely of zeros correspond to comparisons for which no direct evidence is available. Consequently, these comparisons do not contribute evidence to any other comparison. These columns are removed from the table to improve readability.

	aca:met	aca:pla	aca:sul	ben:pla	met:pio	met:pla	met:ros	met:sul	mig:pla	pio:pla	pio:ros	pla:ros	pla:sit	pla:vil	ros:sul
aca-ben	0.07	0.59	0.34	−1	0.05	0.18	0.02	−0.18	0	0.05	0.01	−0.18	0	0	−0.15
aca-met	0.10	0.54	0.31	0	−0.13	−0.40	−0.04	−0.29	0	−0.01	−0.05	0.04	0	0	−0.01
aca-mig	0.07	0.59	0.34	0	0.05	0.18	0.02	−0.18	−0.91	0.05	0.01	−0.18	0	0	−0.15
aca-pio	0.09	0.56	0.35	0	0.62	−0.16	−0.03	−0.22	0	−3.91	−0.15	0.09	0	0	−0.10
aca-pla	0.07	0.59	0.34	0	0.05	0.18	0.02	−0.18	0	0.05	0.01	−0.18	0	0	−0.15
aca-ros	0.07	0.57	0.36	0	0.04	0.02	0.08	−0.15	0	0.08	0.04	−0.04	0	0	−0.21
aca-sit	0.07	0.59	0.34	0	0.05	0.18	0.02	−0.18	0	0.05	0.01	−0.18	−0.91	0	−0.15
aca-sul	0.06	0.41	0.53	0	−0.03	−0.15	−0.01	0.27	0	−0.04	0	0.21	0	0	0.20
aca-vil	0.07	0.59	0.34	0	0.05	0.18	0.02	−0.18	0	0.05	0.01	−0.18	−0.91	0	−0.15
ben-met	0.02	−0.06	0.03	−1	−0.19	−0.58	−0.11	−0.11	0	−0.14	−0.05	0.23	0	0	0.07
ben-mig	0	0	0	0	0	0	−8.85	0	1	0	0	0	0	0	0
ben-pio	0.01	−0.04	0.03	0	0.47	−0.36	−0.06	−0.04	0	−0.36	−0.15	0.24	0	0	0.03
ben-pla	0	0	0	1	0	0	0	0	0	0	0	0	0	0	0
ben-ros	0	−0.03	0.02	1	0	−0.10	0.01	0.03	0	−0.04	−0.05	0.83	0	0	−0.07
ben-sit	0	0	0	1	0	0	0	0	0	0	0	0	0.91	0	0
ben-sul	−0.01	−0.18	0.20	1	−0.03	−0.33	0.04	0.45	0	0.09	−0.01	0.40	0	0	0.35
ben-vil	0	0	0	1	0	0	0	0	0	0	0	0	0	1	0
met-mig	−0.03	0.06	−0.03	0	0.19	0.58	0.11	0.11	0	0.14	0.05	−0.23	1	0	−0.07
met-pio	−0.01	0	−0.01	0	0.61	0.48	0.18	0.14	0	0.09	0.10	−0.06	0	1	−0.13
met-pla	−0.02	0.06	−0.04	0	0.19	0.58	0.11	0.11	0	0.14	0.05	−0.23	0	0	−0.07
met-sit	−0.02	0.06	−0.03	0	0.19	0.58	0.11	0.11	0	0.14	0.05	−0.23	1	0	−0.07
met-sul	−0.04	0.13	0.16	0	0	0.25	0.08	0.26	0	0.06	0.03	0.17	0	0	0.13
met-vil	−0.02	0.06	−0.03	0	0.19	0.58	0.11	0.11	0	0.14	0.05	−0.23	0	1	−0.07
mig-pio	0.01	−0.04	0.03	0	0.47	−0.36	−0.06	−0.04	0	−0.36	−0.15	0.24	0	0	0.03
mig-pla	0	0	0	0	0	0	0	0	0	1	0	0	0	0	0
mig-ros	0	−0.03	0.02	0	0	−0.10	0.01	0.03	0	−0.04	0.05	0.83	0	0	−0.07
mig-sit	0	0	0	0	0	0	0	0	0	1	0	0	0	0	0
mig-sul	−0.01	−0.18	0.20	0	−0.03	−0.33	0.04	0.45	0	1	−0.09	−0.01	0	0	0.35
mig-vil	0	0	0	0	0	0	0	0	0	0	0	0	0	1	0
pio-pla	−0.01	0.04	−0.03	0	−0.47	0.36	0.06	0.04	0	0.36	0.15	−0.24	0	0	−0.03
pio-ros	−0.01	0.01	0	0	−0.43	0.26	0.13	0.10	0	0.32	0.20	0.05	0	0	−0.09
pio-sit	−0.01	0.04	−0.03	0	−0.47	0.36	0.06	0.04	0	0.36	0.15	−0.24	1	0	−0.03
pio-sul	−0.02	0.15	0.17	0	−0.59	0.03	0.02	0.31	0	0.24	0.14	0.15	0	0	0.32
pio-vil	−0.01	0.04	−0.03	0	−0.47	0.36	0.06	0.04	0	0.36	0.15	−0.24	0	1	−0.03
pla-ros	0	−0.03	0.02	0	0	−0.10	0.01	0.03	0	−0.04	0.05	0.83	0	0	−0.07
pla-sit	0	0	0	0	0	0	0	0	0	0	0	0	1	0	0
pla-sul	−0.01	−0.18	0.20	0	−0.09	−0.33	−0.04	0.45	0	−0.09	−0.01	0.40	0	0	0.35
pla-vil	0	0	0	0	0	0	0	0	0	0	0	0	0	1	0
ros-sit	0	0.03	−0.02	0	0	0.10	−0.01	−0.03	0	0.04	−0.05	−0.83	1	0	0.07
ros-sul	−0.01	−0.16	0.17	0	−0.03	−0.23	−0.07	0.42	0	0.04	−0.06	−0.43	0	0	0.14
ros-vil	0	0.03	−0.02	0	0	0.10	−0.01	−0.03	0	0.04	−0.05	−0.83	0	1	0.07
sit-sul	−0.01	−0.18	0.20	0	−0.03	−0.33	0.04	0.45	0	0.09	−0.01	0.40	−1	0	0.35
sit-vil	0	0	0	0	0	0	0	0	0	0	0	0	−1	1	0
sul-vil	0.01	0.18	−0.20	0	0.03	0.33	−0.04	−0.45	0	0	0.01	−0.40	0	1	−0.35

(S2-1)
